# Non-Alcoholic Fatty Liver Disease and Cardiovascular Comorbidities: Pathophysiological Links, Diagnosis, and Therapeutic Management

**DOI:** 10.3390/diagnostics11040689

**Published:** 2021-04-12

**Authors:** Alexandra Jichitu, Simona Bungau, Ana Maria Alexandra Stanescu, Cosmin Mihai Vesa, Mirela Marioara Toma, Cristiana Bustea, Stela Iurciuc, Marius Rus, Nicolae Bacalbasa, Camelia Cristina Diaconu

**Affiliations:** 1 Clinical Emergency Hospital of Bucharest, 105402 Bucharest, Romania; jichitualexandra@yahoo.com (A.J.); drcameliadiaconu@gmail.com (C.C.D.); 2 Department of Pharmacy, Faculty of Medicine and Pharmacy, University of Oradea, 410028 Oradea, Romania; mire.toma@yahoo.com; 3 Department 5, Faculty of Medicine, “Carol Davila” University of Medicine and Pharmacy, 050474 Bucharest, Romania; alexandrazotta@yahoo.com; 4 Department of Preclinical Disciplines, Faculty of Medicine and Pharmacy, University of Oradea, 410073 Oradea, Romania; v_cosmin_15@yahoo.com (C.M.V.); cristianabustea@yahoo.com (C.B.); 5 Department of Cardiology, Faculty of Medicine, “Victor Babeş” University of Medicine and Pharmacy, 300041 Timisoara, Romania; 6 Department of Medical Disciplines, Faculty of Medicine and Pharmacy, University of Oradea, 410073 Oradea, Romania; rusmariusr@yahoo.com; 7 Department 13, Faculty of Medicine, “Carol Davila” University of Medicine and Pharmacy, 050474 Bucharest, Romania; nicolae_bacalbasa@yahoo.ro; 8 Department of Surgery, “Ion Cantacuzino” Clinical Hospital, 030167 Bucharest, Romania

**Keywords:** non-alcoholic fatty liver disease, cardiovascular comorbidities, pathophysiology, treatment

## Abstract

Non-alcoholic fatty liver disease (NAFLD) has a growing prevalence in recent years. Its association with cardiovascular disease has been intensively studied, and certain correlations have been identified. The connection between these two entities has lately aroused interest regarding therapeutic management. In order to find the best therapeutic options, a detailed understanding of the pathophysiology that links (NAFLD) to cardiovascular comorbidities is needed. This review focuses on the pathogenic mechanisms that are behind these two diseases and on the therapeutic management available at this time.

## 1. Introduction

Non-alcoholic fatty liver disease (NAFLD) is a liver disease that can progress from hepatic steatosis to steatohepatitis and even cirrhosis and hepatocellular carcinoma. From the histopathological point of view, it is characterized by excess storage of macrovesicular fat in hepatocytes. These macrovesicular storages are composed of triglycerides that accumulate in the liver. The process can lead in some individuals to an inflammatory response, which is responsible for steatohepatitis, that leads to fibrosis and, finally, cirrhosis [1]. 

The risk factors for developing NAFLD are metabolic syndrome (central adiposity, hyperglycemia, dyslipidemia, arterial hypertension), weight gain, and insulin resistance/diabetes [2,3].

NAFLD can be diagnosed either by imaging techniques or histological examination. Both techniques should be supported by the exclusion of other causes of fatty liver (viral infections, alcohol consumption, autoimmunity, or certain drugs). 

Regarding imaging techniques, abdominal ultrasonography is the most used because its feasibility and low costs [4,5]. Magnetic resonance imaging (MRI), that measures hepatic fat concentration, can also be used, but it is more expensive [6,7].

The standard diagnostic tool of NAFLD is hepatic biopsy. However, this is an invasive and a more expensive procedure and can also be fraught with sampling error and variability in pathologist interpretation [8]. The histological criteria for diagnosing steatohepatitis include the following:

- >5% macrovesicular steatosis;
- presence of inflammation;
- ballooning hepatocytes with predominantly centro-lobular distribution [9].

Biological findings in NAFLD are high serum triglycerides and low levels of high-density lipoprotein cholesterol (HDL-C). Aspartate aminotransferase (AST) and alanine aminotransferase (ALT) can also be mildly increased, with ALT levels higher than AST, but this is more specific to non-alcoholic steatohepatitis (NASH) [10].

The pooled prevalence of NAFLD globally is 25.24%. The highest prevalence rates have been reported in South and Middle East American countries (30%). Similar NAFLD prevalence has been reported in Europe (24%) [2]. Taking into consideration sex differences, the prevalence is higher in men than women but increases in women after menopause [11].

Traditionally, NAFLD was described as a hepatic manifestation of the metabolic syndrome. Recently, it has started to be recognized as a multisystemic disease, which is involved in the pathogenesis of various conditions, such as type 2 diabetes mellitus, cardiovascular disease, and chronic kidney disease [12]. Correlations between NAFLD and cardiovascular comorbidities, independent of traditional cardiovascular risk and metabolic syndrome (MetS), were also demonstrated. NAFLD is associated with hypertension, atherogenic dyslipidemia, that leads to chronic coronary syndrome and stroke, arrhythmias, increased risk of thromboembolic events, and structural heart disease. 

In order to find out the most recent data regarding NAFLD and its implication in cardiovascular comorbidities, PubMed was the most important used database. Two hundred and thirty articles written between 1985–2021 were elected for this review, considering to be in the topic, English written, and relevant for our research. 

The purpose of this paper is to present in detail the pathophysiological connections between NAFLD and cardiovascular diseases, in order to raise physicians’ awareness about the large number of cardiovascular complications (arterial hypertension, coronary artery disease, atrial fibrillation and thrombosis) that a NAFLD patient can be affected by, for a more complete diagnosis of these patients and possibility for early intervention. The article presents the current therapeutic options for NAFLD, aimed to result in NAFLD regression but also, if possible, in cardiovascular risk reduction. Moreover, the review insists on intimate details of NAFLD correlation with components of MetS (such as insulin resistance, altered vasodilatation, pro-inflammatory state, gut dysbiosis, elements that impact cardiovascular health). A better understanding of these mechanisms will lead to an earlier diagnosis of cardiovascular complications in NAFLD patients. 

## 2. Non-Alcoholic Fatty Liver Disease and Arterial Hypertension

Arterial hypertension affects nearly 30% of the adults and is a result of the combination between genetic predisposition and environmental risk factors [13]. Hypertension has long been demonstrated to be associated with NAFLD, studies revealing a high prevalence of NAFLD in hypertensive patients, even without the risk factors for liver steatosis [14]. Although numerous research data show that NAFLD is an independent risk factor for hypertension, all have limitations regarding the techniques used for NAFLD diagnosis, the type of study (e.g., cross-sectional), the use of relative small scales, or they miss some diseases from patients’ history, by gathering information from medical interviews [15,16,17,18]. Taking all this information into account, the question to be answered is what the pathogenic mechanisms behind this association are.

There are many theories that explain how NAFLD can induce hypertension, but most of them have been studied on experimental animals, and the extent of these that can be applied to humans needs further investigations. These theories are systemic inflammation, insulin resistance, increased vasoconstriction and decreased vasodilation, arterial stiffness, increased oxidative stress, gut dysbiosis, and genetic and epigenetic modifications.

### 2.1. Systemic Inflammation 

Studies have shown that NAFLD strongly associates with increased levels of inflammatory cytokines, such as tumor necrosis factor α (TNF-α), interleukin 6 (IL-6), and C-reactive protein (CRP) [19]. In addition, these cytokines are elevated in patients with primary hypertension [20]. 

Liver is an innate immune organ and the continuing production of metabolites, in this case triglycerides that accumulates in hepatocytes, leads to excessive inflammatory cytokines release, that promotes hepatic steatosis and fibrosis [13,21].

Other mechanisms that are involved in the activation of systemic inflammation are increased release of very-low-density lipoproteins (VLDL) from the overloaded triglyceride hepatocytes that stimulates Toll-like receptors (TLRs) and increased levels of hepatokines (fetuin A and retinol-binding protein 4) [22,23]. 

The innate immune components and TLRs are playing important roles in the induction and sustenance of hypertension. 

Another mechanism that is involved in the development of hypertension is inflammation-induced activation of the renine-angiotensin system (RAS). TNF-α and IL-6 are responsible for the regulation of RAS components, especially angiotensinogen production in the liver and kidney, promoting angiotensin-related hypertension. 

Moreover, various clinical evidence has demonstrated the correlation between NAFLD and chronic kidney disease, which is also a cause of hypertension.

In conclusion, these studies contribute to the theory that inflammation that appears in NAFLD is a cause of hypertension that accompanies this affliction [24].

### 2.2. Insulin Resistance 

The mechanisms that are behind insulin resistance (IR) and hypertension are the activation of sympathetic nervous system and the renal sodium retention [25]. IR in NAFLD might be caused by a variety of factors, such as: hepatokines secretion, cytokines, and farnesoid X receptors (FXR). 

Hepatokines originate from the fatty liver. They are able, through VLDL to alter the fatty acids metabolism. Moreover, they induce inflammation and insulin resistance in other cell types [26]. IR itself, is also responsible for the development of NAFLD [27]. Insulin is responsible for vasodilatation through nitric oxide (NO) production, and in reverse, IR stimulates vasoconstriction, leading also to hypertension. 

FXR are identified as a bile acid activated nuclear receptors. They control lipid metabolism by regulating the synthesis, conjugation and transport of the bile acids. In NAFLD, their number is increased and they contribute to the suppression of hepatic FXR-mediated metabolic signaling, which further promotes IR [28].

All of the above considered, it can be stated that IR is, as most, a part of the mechanisms that induce hypertension in NAFLD patients, as it is one of the causes that leads to fatty liver disease.

### 2.3. Increased Vasoconstriction and Decreased Vasodilation

Asymmetric dimethylarginine (ADMA) is a recently discovered marker of endothelial dysfunction and atherosclerosis. It is generated by breaking down the proteins that were post-translationally methylated at an arginine residue [29]. ADMA is endogenously responsible for the inhibition of nitric oxide synthase (NOS), therefore causing the inhibition of vasodilatation. It has been observed that ADMA levels are significantly higher in patients with NAFLD (liver being the main site of elimination), even without traditional cardiovascular risk factors [29]. In addition, there is evidence that ADMA associates with endothelial dysfunction in patients with hypertension [30]. Several studies have shown that circulating ADMA is increased in subjects with histological-proven NAFLD: one study had only male subjects enrolled, with no morbid obesity, type 2 diabetes mellitus (T2DM), or hypertension, and another one showed that ADMA levels are higher in insulin resistance states [31] and that plasma ADMA levels are decreasing as a response to insulin sensitivity improvement [29,32]. Although several studies have demonstrated the correlation between vasoconstriction, ADMA and NAFLD, further investigation still needs to be done given the heterogeneity of the current studies.

### 2.4. Arterial Stiffness 

Arterial stiffness results as a consequence of the complex interaction between stable and dynamic effects in cellular and structural components of the vascular wall [33]. The resistance and compliancy of the arteries are determined by two main structural components: elastin and collagen. Their quantity is kept stable by the interplay between production and degradation. Disruption of this balance, stimulated by an inflammatory context, might lead to reduced quantities of normal elastin and overproduction of altered collagen, therefore leading to increased arterial stiffness [34]. One theory that comes into the support of arterial stiffness and NAFLD is inflamed visceral adipose tissue. In this case, NASH might be responsible for releasing inflammatory, pro-thrombotic and oxidative-stress substances and NAFLD interferes with insulin resistance and atherogenic dyslipidemia [35,36,37].

Several studies, most of them with cross-sectional designs [38,39,40,41] confirmed the connection between increased arterial stiffness and NAFLD, independently of any additional cardio-metabolic predisposing factors. The weaknesses of these studies were their designs, that allow only mere correlations to be made and that the mainly diagnostic method used for NAFLD was ultrasonography. Ozturk et al. used biopsy for the diagnosis of NAFLD. They concluded that independently of MetS, NAFLD is responsible for increasing the risk of atherosclerosis and impaired function of the endothelium in adult male subjects [42]. 

In resume, the possible biological mechanisms that link NAFLD to increased arterial stiffness remain still unknown, but they possibly involve adipokines imbalance and chronic low-grade inflammation [33,43].

### 2.5. Increased Oxidative Stress 

Oxidative stress reflects a disparity between the availability of reactive oxygen species (ROS) and cellular antioxidant system. This imbalance leads to the alteration of the cell functions and eventually cellular death [44]. These ROS are produced by the body due to normal intracellular metabolism and have physiological roles at low concentration, but, in high concentration, they can damage deoxyribonucleic acid (DNA) [45]. NAFLD has been suggested to be linked to oxidative stress [13,46].

One major source of oxidative stress is represented by homocysteine and the liver is a major metabolic organ for this. Alterations of the homocysteine metabolism involves impaired re-methylation to methionine and reduced trans sulphuration to cysteine. Therefore, intrahepatic vascular resistance is increased due to impaired NO formation, as homocysteine reduces NO release from sinusoidal endothelial cells and also causes hepatic stellate cell contraction [47]. Moreover, the production of ROS in NAFLD is responsible for the oxidation of low-density lipoproteins (LDL), which induces the transformation of the macrophages into foam cells. This is the first step of the atherosclerotic lesion development [48].

All these particularities described above suggest that oxidative stress is in charge for both increased intrahepatic resistance and atherosclerosis. In addition, clinical evidence has shown that elevated homocysteine levels are associated with hypertension [49].

### 2.6. Gut Dysbiosis 

Axenic animal models have long been used to assess the repercussions that the absence of gut microbiota have. 

Regarding NAFLD, it has been observed that the absenteeism of intestinal flora in mice leads to the gathering of active constituent ligands/androstane receptor (CAR), bile acids, bilirubin, and steroid hormones, which lead to alteration of hepatic xenobiotic metabolism, which could favor the development of NAFLD [50]. The mechanisms that are involved in the correlation between gut microbiota and NAFLD are changes in the quantity of energy absorbed from food, changes in the permeability of the intestine, and alterations of the expression of the genes involved in the lipogenesis. In addition, choline and bile acid metabolic signaling pathways are involved mechanisms, along with the production of ethanol in the intestine and interactions with the innate immunity [51]. 

On the other hand, gut microbiota and, specifically, dysbiosis has an important role in hypertension development [13]. Regarding the effect that dysbiosis has on hypertension, the following mechanisms are involved: inflammation, vasodilatation, and short-chain fatty acids. Intestinal dysbiosis regulates the differentiation and maturation of immune cells, therefore being implied in the systemic inflammatory state [52].

Vasodilatation is a mechanism that protects against hypertension. It seems to be caused by short-chain fatty acids in the presence of specific gut microbes, a process which is mediated through G-coupled protein receptors. The intestinal flora of the patients suffering from prehypertension and hypertension had a larger amount of Prevotella, a type of enteric bacteria, whereas the intestinal flora of controls individuals contained mostly bacteria from the genus Bacteroidetes [52].

So, it can be assumed that there is a correlation between hypertension, NAFLD, and gut dysbiosis.

### 2.7. Genetic and Epigenetic Modifications 

Although there are consistent data that prove the link between hypertension and NAFLD, genetics research is limited regarding this association [13]. Family studies demonstrate that first degree relatives of the NAFLD patients have a much higher risk of developing the disease than the general population, and same familial distribution has long been proved for primary hypertension [53].

*Adiponectin* (ADIPOQ) gene (which encodes adiponectin) polymorphisms have been indicated to be the link between hypertension and NAFLD [54]. Adiponectin is a hormone derived from adipocytes. It has an important role in modulating lipid and glucose metabolism [55].

Another gene that was investigated was angiotensin receptor type 1 (AGTR1). In a recent prospective cohort study, the gain-of function *A1166C* (*rs5186*) variant in the *AGTR1* gene represented a strong predictor for incident NAFLD and associated hypertension. In addition, polymorphism in AGTR1 may influence the risk of liver fibrosis in NAFLD [56]. 

Regarding the epigenetic changes, they are responsible for the interaction with inherited risk factors and, therefore, determine the susceptibility of NAFLD, hypertension, and cardiovascular disease [57,58].

Although promising research data show a clear link between hypertension and NAFLD, considering genetics and epigenetics, many data are still missing and more detailed investigations should be carried out.

## 3. Non-Alcoholic Fatty Liver Disease and Coronary Heart Disease

Several studies have proven the link between NAFLD and coronary artery disease (CAD). Most of them have demonstrated a correlation between hepatic steatosis and the calcification of coronary arteries [59,60]. 

In a meta-analysis, Ampuero et al. evaluated the impact of NAFLD on subclinical atherosclerosis and CAD. 14 studies were reviewed. CAD was taken into consideration when patients showed ≤50% stenosis in one or more of the major coronary arteries. Hepatic biopsy and ultrasound were used to evaluate NAFLD. Outcomes revealed that the intima-media thickness of carotid in patients with NAFLD had a higher prevalence (35.1% vs. 21.8%). On the other hand, four studies that assessed CAD by coronary angiogram showed that 80.4% of the subjects with NAFLD had CAD, while only 60.7% of the patients without NAFLD had CAD. The conclusion was that NAFLD increases the risk of coronary artery disease [61].

Another cross-sectional study, carried out by Chang et al., evaluated coronary artery calcification and its relationship with alcohol-induced fatty liver disease and (NAFLD). One hundred five thousand three hundred and twenty-eight Korean adults were included. Coronary artery calcium (CAC) score was assessed using computed tomography, liver fat by ultrasound and alcohol intake by g/day. The authors came to the conclusion that both alcohol-induced fatty liver disease and NAFLD were firmly associated with CAC score, without significant interaction with obesity [62].

Besides the fact that the relationship between coronary heart disease and NAFLD was strongly investigated, and that the correlation is clear, the pathophysiology and mechanisms that are behind still need to be investigated. Currently, it is thought to be a complex process that involves insulin resistance, adipokines, oxidative stress, and apoptosis [63].

### 3.1. Insulin Resistance

This theory is supported by various studies demonstrating that the higher the liver fat content is the lesser the hepatic insulin sensitivity is.

Kotronen et al. carried out a study where T2DM patients were examined. They concluded that insulin resistance in patients with increased liver fat is due to diminished insulin clearance. Sixty-eight patients with T2DM were compared to a control group containing non-diabetic subjects. Liver steatosis was determined by proton magnetic resonance spectroscopy. The clearance and action of insulin was assessed by the infusion of (3-3H) glucose and by the euglycemic insulin clamp technique. In addition, MRI was used in order to determine the body composition. The results confirmed that type 2 diabetic patients had higher liver fat (54%) and lower insulin clearance (24%) than nondiabetic subjects. In addition, their insulin levels were higher (34 mU/L vs. 25 mU/L) [64].

Jun N-terminal kinases (JNKs) are kinases involved in the survival and differentiation of the cells. They belong to the mitogen-activated protein kinase (MAPK) superfamily [65]. JNKs are involved in insulin resistance and have important roles regarding the β-cells of the pancreas. Those roles are concerning their secretory function and survival. They are activated by inflammatory cytokines. It has been found that insulin resistance is associated with the formation of autophagosomes in pancreatic β-cells [66]. The main mechanisms that are proposed to be responsible for the alteration of the insulin signaling molecules are post-transcriptional modifications. Various kinases are able to phosphorylate the substrate of the insulin receptor. This phosphorylation is responsible for the inhibition of this receptor [67]. TNF- α is a proinflammatory cytokine effector that is over-expressed in obese patients. It can activate JNKs and, therefore, mediate IR by inhibiting insulin signaling in the liver [68]. 

On the other hand, chronic high concentrations of glucose and leptin induce the secretion of IL-1β from the pancreatic islet, that can also possibly promote β-cell malfunction and death. This mechanism is also mediated by the JNK pathway. Therefore, the inhibition of JNK might protect the pancreatic β-cells from the outcomes that high levels of leptin and glucose have in diabetic patients.

Another reason that leads to insulin resistance is chronic oxidative stress. This mechanism is linked to JNKs that can mediate the consequences that stress has on insulin resistance. This mediation is due to the inhibition of the phosphorylation of insulin receptor substrate 1 (IRS-1). In addition, free fatty acids are able to activate, via JNKs, autophagy in pancreatic β-cells.

### 3.2. Adipokines 

Studies have shown that adipokines (cytokines secreted by the adipocytes) are increased in NAFLD patients. 

Yilmaz et al. investigated the patients with histological confirmed NAFLD regarding the relationship between the thickness of epicardial fat (EFT), adipokines related to epicardial fat, and coronary flow reserve (CFR). Fifty-four subjects with NAFLD (26 males and 28 females) and 56 control subjects (27 males and 29 females) were analyzed. NAFLD was evaluated by endoscopic ultrasound-guided biopsies. CFR and EFT were assessed by transthoracic cardiac ultrasonography. In addition, enzyme-linked immunosorbent assay (ELISA) was used in order to measure serum levels of vaspin and chemerin. The results proved that CFR was notably lower and EFT significantly higher in subjects with fatty liver disease than in controls. In addition, patients with NAFLD had serum levels of chemerin and vaspin significantly increased compared to controls [69]. 

Some examples of adipokines are leptin, adiponectin, resistin, and TNF-α. Because adipokines are involved in insulin resistance and inflammation, they might also be involved in NAFLD pathogenesis. TNF-α intercedes with insulin signaling and has also proinflammatory action. It has a major role in the apoptotic and proinflammatory responses to endotoxin [70]. Adiponectin is a protein that has completely opposite effects to TNF-α and also suppresses its secretion [71]. Regarding the lipidic metabolism, adiponectin has anti-lipogenic effects and reduces fat deposition. These effects are due to the inhibition of hepatic gluconeogenesis and also to the suppression of lipogenesis [72]. It has been shown that patients with NAFLD have hypoadiponectinemia, which is linked to CAD and diminished glucose tolerance in non-diabetic individuals [73,74].

### 3.3. Oxidative Stress 

The factors that are proposed to be responsible for oxidative stress are hyperinsulinemia, bacterial overgrowth, hepatic iron, and lipid peroxidation [75]. 

Bacterial overgrowth might increase the hepatic oxidative stress by increasing the endogenous manufacturing of ethanol. Moreover, other mechanisms that may be involved in this process are the activation of inflammatory cytokines and macrophages [76].

It has been shown that patients that undergo chronic peritoneal dialysis only develop hepatic steatosis when insulin is infused to the peritoneal fluid dialysis [77,78]. This effect might be due to the ability of insulin to generate oxidative stress [79]. Another explanation is that insulin has the ability to up-regulate the lipogenic protein, sterol regulatory element-binding protein (SREBP) [80]. On the other hand, recent studies show that insulin might also be involved in apoptosis. This process might be due to insulin’s capacity to cause stress to endoplasmic reticulum, which leads to unfolding protein response [81]. 

Regarding iron’s prooxidant role, its contribution is still unclear. The only data that comes into the support for its oxidant role suggests that almost 30% of the NAFLD patients have elevated ferritin [82,83,84]. In addition, it has been reported that phlebotomy improves hepatic histology in patients with NAFLD [85]. Further investigations still need to be carried out regarding iron’s role in NAFLD.

### 3.4. Lipid Peroxidation and Apoptosis 

The starting point of oxidative stress within the liver in patients with fatty liver disease are the free fatty acids (FFAs). Elevated FFAs act as ligands for peroxisome proliferator-activated receptor, which is in charge of the up-regulating of FFAs oxidation. This process take place in mitochondria, microsomes, and peroxisomes. FFAs oxidation results in hydrogen peroxide, superoxide, and lipid peroxides that generate oxidative stress and consequent lipid peroxidation [86].

Apoptosis is a mechanism that has multiple pathophysiologic causes and takes part in the development of liver injury and steatosis [75]. It affects hepatocytes and natural killer T cells. The causes of apoptosis in NAFLD are increased TNF, hyperinsulinemia, and oxidative stress. TNF serum levels are higher in patients with NAFLD and obesity. It is responsible for increased mitochondrial permeability, impaired mitochondrial respiration, and depleted mitochondrial cytochrome, all mechanisms leading to apoptosis [87,88]. 

As it was discussed before, hyperinsulinemia is another mechanism responsible for apoptosis. This effect might be due to insulin’s capacity to generate oxidative stress or up-regulate the lipogenic protein, SREBP [79,80]. On the other hand, hyperinsulinemia might also have direct effects on fibrogenesis by increasing the activity of connective tissue growth factor, mainly if hyperglycemia is present [89]. 

Regarding oxidative stress, malondialdyde (MDA) and hydroxynonenal (HNE) are two of the byproducts. They are able to attract neutrophils, to act as stimulants for hepatic stellate cells and to increase the number of receptors for cytokines and transforming growth factor-β (TGF-β) in macrophages [90]. Oxidative stress can also stimulate the discharge of TNF from the hepatic cells, lipocytes, and macrophages [90]. Hepatic stellate cells have the capacity to engulf the apoptotic bodies, a process that might stimulate their fibrogenic process [91]. Another mechanism that is responsible for hepatocyte deaths is ROS-induced fatty acid synthase (FAS) ligand [92,93].

In conclusion, there is a direct connection between NAFLD and CAD. Despite the fact that the pathophysiologic mechanisms behind this correlation still need further investigation, it is clear that a multidisciplinary approach of these patients must be taken into consideration.

## 4. Non-Alcoholic Fatty Liver Disease and Cardiac Arrhythmias

### 4.1. Atrial Fibrillation 

In the OPERA (Oulu Project Elucidating Risk of Atherosclerosis) prospective study, 958 hypertensive subjects and control subjects of the same age and sex were arbitrarily selected from the national registries in 1990s, and the association between atrial fibrillation (AF) and NAFLD was determined. NAFLD was diagnosed by sonography and AF was tracked in the National Registers. The aim of this research was to evaluate the capacity of NAFLD to predict AF. The subjects included in this study were followed-up for 16.3 years. The authors concluded that NAFLD was an independent predictor of AF [94]. Another prospective study carried out by Giovanni et al. followed 400 patients with T2DM over 10 years. The subjects had no AF to start with. They also found out that NAFLD is related to a higher incidence of AF [95].

### 4.2. Ventricular Arrhythmias, Bundle Branch, and Atrioventricular Blocks

Recent data have suggested that type 2 diabetes patients with NAFLD have a higher chance of heart rate-corrected QT (QTc) interval prolongation. QT prolongation is a known risk factor for ventricular arrhythmias and sudden cardiac death.

Hung et al. carried out a cross-sectional study that included 31,116 participants. QTc interval was derived from Bazett’s formula and from electrocardiography. Ultrasonography was used to diagnose and classify NAFLD. NAFLD was staged as severe, moderate, mild, or none. The analyses found that mild, moderate, and severe NAFLD were associated with increased QTc interval compared to no NAFLD. The conclusions were that NAFLD was indeed associated with an increased risk of prolonged QTc interval among general population, independently of the presence of diabetes [96]. 

Another study carried out by Mantovani et al. has retrospectively evaluated 330 patients with T2DM and no end-stage renal disease, preexisting atrial fibrillation, or known liver diseases. The patients undergone 24-h Holter monitoring between 2013 and 2015. NAFLD was diagnosed by ultrasonography. Their study concluded that NAFLD was linked to a higher risk of prevalent ventricular arrhythmias in subjects with T2DM [97].

Matovani et al. carried out a retrospective study assessing whether there is any association connecting fatty liver disease and heart blocks. Seven hundred and fifty-one patients with T2DM were examined during 2007–2014. Atrioventricular blocks were assessed by electrocardiogram and NAFLD was diagnosed by ultrasonography. The study concluded that patients with fatty liver disease had a much higher risk of persistent heart block than those without NAFLD [98]. 

### 4.3. Mechanisms Behind Non-Alcoholic Fatty Liver Disease and Cardiac Arrhythmias 

Currently, it is difficult to ignore the possibility that similar risk factors might be the cause behind cardiac arrhythmias and NAFLD. Obesity is generally associated with NAFLD and AF development [99,100]. On the other hand, both obesity and NAFLD are strongly associated with the accumulation of fat in the epicardium. Epicardial fat is linked to an increased mass of the left ventricle and diastolic dysfunction, the latter being associated with the promotion of AF [101]. Recent studies link NAFLD and diastolic dysfunction. The mechanisms behind this association might be extensive toxic results, mediated through adiponectin, insulin, and inflammation or indirect effects of hypertension or diabetes [102].

On the other hand, NAFLD is also associated with autonomic nervous system dysfunction and so is epicardial fat, dysfunction that is a risk factor for AF [103,104].

**Figure 1:**
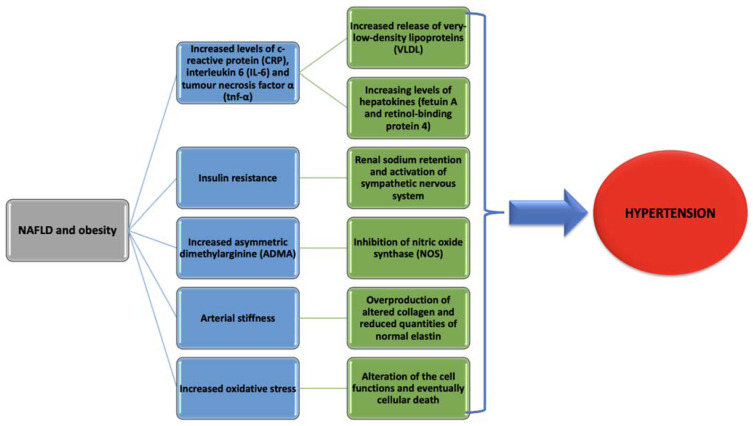
Pathogenic mechanisms behind hypertension development in non-alcoholic fatty liver disease (NAFLD) and obesity.

Regarding systemic inflammation, there are studies revealing that AF is as most a cause of systemic inflammation as it is a consequence [105], and, as it was discussed before, NAFLD is also associated with systemic inflammation. Moreover, hypoadiponectinemia, a usual finding in NAFLD is also related to AF [106]. This might explain how obesity and inflammation seem to play an important part in the relationship between NAFLD and AF. Taking all this information into consideration, we are still unable to differentiate if NAFLD is a cause of AF without the association of metabolic syndrome or if AF can itself contribute to the progression of NAFLD. Therefore, further studies are still needed in order to evaluate the real contribution of NAFLD to AF. Figure 1 presents the way that NAFLD and obesity increase the risk of developing hypertension.

One study carried out by Lin YK et al. analyzed how adipocytes modulate the electrophysiology of atrial myocytes [15]. They used whole-cell patch clamp in order to record the action potentials and ionic currents in myocytes coming from rabbit left atrium. Myocites were incubated with and without adipocytes belonging to different sites of the body or adipocytes-conditioned supernatant for 2–4 h. Compared to control left atrium myocytes, left atrium myocytes incubated with adipocytes had longer action potentials durations. Left atrium myocytes incubated with adypocites from the epicardium had a more positive resting membrane potential than control left atrium myocytes. However, left atrium myocytes that were incubated with supernatant had longer action potential durations but similar resting membrane potential compared to control myocytes. Moreover, isoproterenol induced a higher incidence of provoked heart beats in left atrium myocytes that were incubated with adipocytes compared to the control group myocytes. All these taken into consideration, they concluded that adipocytes are able to cause arrhythmic heart beats in the myocytes of the left atrium. This might be possible due to adipocytes’ capacity to modulate electrophysiological characteristics and ion currents [107].

Moreover, pro-inflammatory cytokines and protrombotic factors in NAFLD are also linked to increased cardiac structural and arrhythmogenic complications [108].

On the other hand, NAFLD has been shown to be involved in the modifications of the myocardium functions and structure [109]. These modifications may induce fiber discontinuity and circuit re-entry, leading to electrophysiological disturbance [110].

In closing, it might be stated that there is indeed a powerful association between NAFLD and cardiac arrhythmias. Whether the common risk factors of these two entities are the cause or whether NAFLD is itself responsible for cardiac arrhythmias is still poorly investigated and further studies need to be carried on.

## 5. Non-Alcoholic Fatty Liver Disease and Altered Cardiac Structure 

Multiple studies have reported the coexistence of NAFLD and altered cardiac structure. These modifications, regarding heart involve:

- abnormalities in cardiac metabolism;
- increased left ventricular mass;
- increased interventricular septum thickness;
- diastolic cardiac dysfunction;
- left atrium enlargement or impaired left atrium deformation;
- decreased right ventricular function;
- aortic and mitral valves calcification;
- congestive heart failure [111].

### 5.1. Abnormalities in Heart Metabolism 

Perseghin et al. carried out a study assessing if subjects with fatty liver disease have also modifications regarding the left ventricle (LV). The modifications studied where those concerning the amounts of epicardial fat, the structure, energy metabolism, and function. Forty-two young, non-diabetic men, including 21 with NAFLD and 21 without NAFLD, were investigated. Cardiac MRI, cardiac 31phosphorus-magnetic resonance spectroscopy (31P-MRS) and hepatic 1Hydrogen-MRS (1H-MRS) were used as diagnostic tools. The updated Homeostasis Model Assessment (HOMA-2) computer model was used to determine insulin sensitivity. Systolic and diastolic functions, as well as left ventricular morphology, were not different among the two groups. On the other hand, the authors found out that intrapericardial and extrapericardial adipose tissue was increased in male subjects with fatty liver disease correlated to controls. The phosphocreatine (PCr)/adenosine triphosphate (ATP) ratios is an in vivo marker of myocardial energy metabolism. These ratios were reduced in men with NAFLD. Therefore, they concluded that although the morphological features and systolic and diastolic functions of the LV were normal, the energy metabolism was altered [112]. 

Despite the studies that show consistent evidence regarding altered heart metabolism, the pathogenic mechanism behind this is ambiguous. Ectopic liver fat accumulation is thought to be due to the increased free fatty acids flux, as it was showed before. Regarding the myocardium, it is known that its main source of energy in the fasting state are FFAs [113]. When the supply of FFAs outreaches the rate of their oxidative disposal, as happens in NAFLD, oxidative stress is induced, with the consequence of increased lipid intermediates and lipotoxicity. Lipotoxicity may be involved in the impairment of energy homeostasis and contractile dysfunction [114]. Other theories that might explain this perturbation, regarding the heart metabolism are the involvement of adipokines and inflammation. Because none of these hypotheses could be confirmed by this study, insulin resistance was thought to also be involved. 

Concluding, although strong association between abnormalities in the heart metabolism and NAFLD exists, further studies providing information regarding the pathophysiology still need to be carried out.

### 5.2. Increased Left Ventricular Mass and Interventricular Septum Thickness

In a recent study, Hayrullah et al. assessed the grades of NAFLD and the cardiac functions and associated parameters [115]. Four hundred obese children participated in the study. Ultrasonography was used in order to diagnose NAFLD. Ninety-three children had NAFLD, 307 were in the non-NAFLD group, and these two subgroups were compared to 150 control subjects. In addition, pulsed and tissue Doppler echocardiography were used, and intima-media and epicardial adipose tissue were measured. Fatty liver disease subgroups had increased end-systolic thickness of the interventricular septum and larger left ventricular mass. Moreover, carotid artery intima-media and epicardial adipose tissue thickness were higher in obese children.

One pathologic mechanism that might explain these modifications is the eccentric remodeling of the LV as an effect of adaptation to volume overload. Moreover, central fat distribution is associated to higher cardiac output [116]. 

On the other hand, it is established that insulin resistance increases the chances for LV dysfunction development. Obese patients have increased levels of insulin and altered insulin sensibility, which is also related to NAFLD, as it was discussed before. Elevated insulin levels stimulate the growth of myocytes and interstitial fibrosis, by sodium retention and activation of sympathetic nervous system [117]. 

A meta-analysis carried out by Borges et al. noted an important relationship between NAFLD and higher left atrium diameter and ratio between left atrial volume and body surface area [118]. The left atrium is responsible for the filling of the left ventricle, and, on the other hand, the left ventricle function influences the left atrium’s function throughout the cardiac cycle [119]. As a consequence of eccentric remodeling of the LV, LV’s filling pressure increases and leads to left atrium dilatation. Therefore, left atrium remodeling occurs and atrial compliance and contractile functions decrease [120].

### 5.3. Decreased Right Ventricular Function

Bekler et al. studied systolic and diastolic function of the right ventricle and its association with NAFLD [121]. Thirty-two patients had NAFLD diagnosed by ultrasonography, and 22 represented the control group, i.e., had no NAFLD. The function of the right ventricle was assessed using conventional and tissue Doppler echocardiography. Right ventricle global function was evaluated by myocardial performance index (MPI). They concluded that right ventricular diastolic dysfunction correlated with fatty liver disease and the level of fatty changes. 

Regarding this association, there are speculations assuming that the promotion of right ventricular diastolic function is due to the excess of lipid accumulation in hepatocytes, that leads to lipid deposition in cardiac myocytes.

Aortic and mitral valves calcification: Several studies have demonstrated that NAFLD associates with calcium deposits of the aorta and mitral valves [122].

Aortic valve sclerosis is associated with a higher incidence of cardiovascular disease mortality. This statement is valid for both diabetic and non-diabetic patients. In addition, the calcium deposits in the mitral valve are associated with adverse cardiovascular disease outcomes [123]. One mechanism that may explain the link between NAFLD and the calcification of aortic and mitral valves is chronic inflammation. It was discussed in the previous paragraphs how the liver produces inflammatory markers. These are believed to contribute to the acceleration of atherosclerosis, as it is demonstrated in histopathological examinations, which show that valve sclerosis is an inflammatory condition mixed with classic atherosclerotic lesions [124,125]. 

In addition, insulin resistance might be responsible for the association between hepatic steatosis and aortic valve sclerosis. One small cross-sectional study concluded that increased steatosis of the liver is associated with lower insulin clearance, which contributes to insulin resistance in non-diabetic subjects [126]. However, another study carried out by Marcello et al. [127] provided evidence that clinical diabetes mellitus did not substantially affected the results. 

### 5.4. Diastolic Cardiac Dysfunction and Risk of Congestive Heart Failure

Explanations behind diastolic cardiac dysfunction and NAFLD are systemic inflammation, insulin resistance and increased prevalence of metabolic disorders. 

Systemic inflammation is mediated by NAFLD, as it was discussed before. Cytokines are able to modify the structural substrate and electrophysiology of the myocardium. Moreover, this pro-inflammatory condition is associated with inflammation in the coronary microvascular endothelium. This is able to increase the LV diastolic stiffness and lead to cardiac failure [128].

Insulin resistance, a common comorbidity among patients with NAFLD and obesity, alters diastolic function. This is possible due to insulin’s ability to regulate the expression of myosin gene [129]. 

Metabolic disorders, such as hypertension, are risk factors for diastolic cardiac dysfunction and are frequently associated with NAFLD.

Taking all the information above into consideration, it is likely to assume that fatty liver disease is possibly linked to a higher chance of developing congestive heart failure. Several researches that used elevated aminotransferases levels or serum gamma-glutamyl transferase (GGT) as markers of NAFLD have proven that this disorder is strongly connected to heart failure [130,131].

Regarding GGT, the mechanisms that may speculatively be involved in this association are the presence of GGT in atherosclerotic plaques and that elevated GGT levels might be a marker of increased oxidative stress [132].

## 6. Non-Alcoholic Fatty Liver Disease and Stroke

Multiple studies have been evaluating over the years the correlation between NAFLD and the presence of stroke.

Hamaguchi et al. have prospectively analyzed 1637 apparently healthy patients. NAFLD was determined by ultrasound. After five years of assessments, a self-administered questionnaire was used to evaluate the incidence of cardiovascular disease. Between the 1221 subjects in attendance for results analysis, the occurrence of cardiovascular disease was elevated in the group composed of the subjects that had NAFLD to start with than the group without NAFLD [133]. 

Another prospective study carried out by Abdeldyem et al. evaluated the anticipating value that NAFLD has on stroke gravity and prognostic. Two hundred subjects that suffered an acute ischemic stroke were studied. Regarding NAFLD, the diagnosis was based on the elevated levels of serum aminotransferases and the lack of any other causes that are able to increase them. They concluded that NAFLD was associated with acute ischemic stroke in 42.5% of the subjects. Moreover, NAFLD might also be related to even worse gravity and prognostic of stroke [134]. 

Regarding the pathophysiology of the correlation between NAFLD and stroke, evidence suggests a relationship between ischemic stroke and biological markers of NAFLD [135]. These biological markers are represented by AST, ALT, and γ-glutamyltransferase (GGT).

A study by Bots et al. analyzed how GGT, as a marker of alcohol consumption associated with hemorrhagic, ischemic, fatal and non-fatal stroke. Three European cohort studies were included, taking part in EUROSTROKE. They concluded that an increased GGT level was linked to an increased chance of developing hemorrhagic stroke [136]. As it was previously discussed, GGT is an enzyme that is presented in the atherosclerotic lesions. GGT locates in the CD68 macrophage-derived foam cells [137]. In addition, other evidences show that GGT is identified in the circulating platelets and granulocytes [138,139]. On the other hand, GGT seems to be adsorbed into circulating LDL. The main role of GGT is the degradation of glutathione, which is an important antioxidant. In selected conditions, GGT plays a prooxidant role. Moreover, it has been demonstrated that GGT-mediated cleavage of glutamate-cysteine-glycine (GSH) can affect the reduction of ferric iron to ferrous iron, this process being able to produce reactive oxygen species [140]. Oxidative reactions are important determinants of the plaque development and instability [141]. They are able to regulate matrix metalloproteinases, which are key factors in determining the plaque stability. 

After all these presented before, it can be stated that GGT, which is an important constituent of the atherogenic plaques, might be a major pro-oxidant component and also a marker of stroke.

## 7. Non-Alcoholic Fatty Liver Disease and Thromboembolic Events

Recent studies proposed that NAFLD patients have an increased chance of developing thromboembolic events regardless of the presence of diabetes, hyperlipidemia, and obesity that are usually associated to NAFLD. Although there are few statistical analysis concerning the associations between fatty liver disease and thromboembolism, recent studies have reported a higher risk of portal venous and systemic thrombosis in forms of advanced NAFLD disease [142].

A case-control study carried by Di Minno et al. documented idiopathic venous thromboembolism (VTE) in 138 patients, with 276 subjects being used as controls. All of them underwent clinical/laboratory/ultrasound evaluation for the presence of metabolic syndrome and NAFLD. One hundred and twelve out of 138 cases and 84/276 controls were diagnosed with VTE. Therefore, they concluded that NAFLD was independently associated with idiopathic VTE [143].

Stine et al. used a cross-sectional research for the evaluation of autonomous association between NASH cirrhosis and portal vein thrombosis (PVT) in patients who experienced hepatic transplantation. T\hirty-three thousand three hundred and sixty-eight patients were included in the study. Of these, 6.3% had PVT. Among them, 12.0% had NASH. When these subjects were compared to another group with different causes of cirrhosis, it was found that the subjects that underwent transplantation had a higher prevalence of PVT. Ten and one tenth percent were having PVT at the time of transplantation, compared to 6.0% without NASH [144].

Metabolic syndrome: As it was discussed before, metabolic syndrome or X syndrome is, as well, a cause, as it is a consequence of NAFLD. X syndrome is a condition that identifies with hypercoagulation, platelet activation and endothelial dysfunction, all those mechanisms being responsible for the occurrence of atherosclerosis. Moreover, metabolic syndrome is also associated with the reduction of anticoagulant proteins and the inhibition of fibrinolysis [145].

Insulin resistance: Insulin resistance in NAFLD is responsible for the increased levels of triglycerides, which is, in turn, responsible for promoting hypercoagulation by elevating the coagulation factors [146].

Moreover, impaired fasting glucose and diabetes are associated with a higher risk of developing cardiovascular diseases and arterial thrombosis [147]. The mechanisms behind this association are thought to be increased thrombin generation, dysfunctional activation of the platelets, calcium release, and inositol phospholipid turnover [148,149]. 

In addition, hyperglycemia is also responsible for altered coagulation cascade by inducing oxidative stress, decreasing plasmatic heparin sulphate and by the non-enzymatic glycation of proteins [150].

Another factor that contributes to the prothrombotic state is NO deficiency, this monoxide being responsible for the inhibition of platelets’ aggregation [151]. As it was discussed before, insulin resistance is responsible for limiting the NO production.

TNF-α, which is increased in NAFLD, impairs the synthesis of endothelial NOS (eNOS). eNOS contributes to a lower amount of NO compared to inducible NOS (iNOS). Several cell types express iNOS, which generates NO as a result of inflammatory state. In this regard, during the inflammatory state, the suppression of iNOS was associated with the diminution of coagulation [152].

Gut microbiota: Studies have shown that a diet rich in fats in mice that develop NAFLD altered the manufacturing of metabolites dependent on intestinal flora, including trimethylamine (TMA) [153]. The relationship within TMA and NAFLD has also been verified in humans [154]. TMA enhances the formation of foam cells from macrophages and platelet activation [155].

Plasminogen activator inhibitor-1 (PAI-1): PAI-1 suppresses tissue plasminogen activator (tPA), therefore reducing the activity of fibrinolysis. NAFLD is linked to higher concentrations of PAI-1, therefore coming into the support of another cause of prothrombotic state [156].

Thromboinflammation, thrombocytes, and activated endothelium: Thrombosis and inflammation are firmly joint. Higher C-reactive protein levels are responsible for the promotion of the interaction between the monocytes and the endothelium and for the activity of PAI-1 and formation of the tissue factor. As a result, the natural anticoagulant systems are down-regulated [157].

NAFLD is associated with a high inflammatory state. This proinflammatory condition is also marked by thrombocytes activation and endothelial impaired function. Endothelial cells produce von Willebrand factor (vWF), factor VIII, and fibrinogen, that are, both, inflammatory and clotting elements.

P-selectin glycoprotein ligand-1 and P-selectin mediate the contact between platelets and endothelium [158]. In addition, adhesion molecules, such as intercellular adhesion molecule one-1 (ICAM-1), are involved in this process, and it has been shown that plasma levels of ICAM-1 are increased in NAFLD [156].

## 8. Therapeutic Management

### 8.1. Lifestyle Management and Dyslipidemia Treatment

All patients with NAFLD, regardless of their weight, should be advised to regularly exercise. Regular exercise improves insulin sensitivity. The types of exercises that are recommended are brisk walking, jogging, or other aerobic exercises for at least 30 min/day, 5 days/week. 

For patients that are overweight or obese, bariatric or weight loss surgery is advised. Initial weight reduction should be 10% of the body weight in 6–8 months [159]. Unfortunately, only marked weight loss can significantly lower both the mass and inflammation of hepatic and epicardial adipose tissue. In addition, marked weight loss has benefits regarding atrial fibrillation, diastolic filling and cardiac insufficiency [160].

Lifestyle management can also improve dyslipidemia. However, for patients with increased cardiovascular risk, additional therapeutic management should be considered and statins are the most attractive group that can be safely applied to patients with NAFLD. Besides their major effects on lowering the LDL concentrations, statins also induce endothelial NO synthase. 

Because statins might increase liver enzymes, in patients that associate high levels of AST and ALT, ezetimibe might have a synergistic effect [161]. Moreover, patients with normal cholesterol levels benefit, as well, from statin treatment. Despite their effect on lowering plasma cholesterol, statins can also reduce the inflammation and maturation of adipose cells, therefore lowering the production of leptin [162].

Statins can also minimize inflammation and mass of adipose epicardial tissue. This effect is responsible for the prevention of myocardial fibrosis, therefore ameliorating the evolution to atrial myopathy [163]. As a result, they are also involved in reducing the development and the recurrence of atrial fibrillation [164,165]. 

Kim et al. investigated the alteration of gastrointestinal microbiome by statin therapy [166]. Rosuvastatin and atorvastatin raised the numbers of Mucispirillum, Bacteroides, and Butyricimonas, which were correlated with the inflammatory response. On the other hand, subjects with high levels of triglycerides might benefit by weight loss, by improving insulin resistance and by using polyunsaturated fatty acids (fish oil) [161].

### 8.2. Diabetes Mellitus Treatment 

Diabetes mellitus is correlated with a higher chance of developing cardiovascular disease; therefore, specific treatment should be taken into consideration [161].

#### 8.2.1. Pioglitazone

Pioglitazone is a particular stimulator of peroxisome proliferator-activated receptor gamma (PPAR-γ) and also a potent insulin sensitizer. As NAFLD independently raises the chances of diabetes mellitus development, and pioglitazone decreases the risk, it was assumed that pioglitazone might also decrease the possibility of developing T2DM in patients with NAFLD. Moreover, pioglitazone lowers the values of arterial hypertension. It is also able to lower the risk of all-cause mortality, myocardial infarction, or cerebrovascular accident in T2DM with macro vascular disease [167,168]. Moreover, pioglitazone improves the development of fibrous tissue in NASH subjects in the absence of diabetes [169].

Regarding gut microbiota, one study carried out by Mei Li et al. revealed that pioglitazone shaped the gut microbiota and ameliorated the natural barrier formed by the enteric epithelium in fructose-fed mice [170]. Given the evidence, it is plausible to assume that pioglitazone could safely be used in the treatment of NASH.

#### 8.2.2. Metformin

Metformin is an oral hypoglycemic agent, used as the first choice of treatment in T2DM. Its effectiveness is due to the ability of reducing blood glucose. This process is accomplished by decreasing liver gluconeogenesis, stimulating muscle glucose uptake, and increasing the oxidation of fatty acids in the adipose tissue, finishing with the augmentation of peripheral insulin responsiveness.

On a molecular degree, its favorable consequences are due to the phosphorylation and nuclear export of liver kinase B1 (LKB1). LKB1 is responsible for the activation of adenosine monophosphate-activated protein kinase (AMPK). AMPK is able to trigger the catabolic pathways that produce adenosine triphosphate (ATP) and to suppress the anabolic mechanisms that consume ATP [171,172]. 

Energy strain in the muscular fibers activates AMPK. Therefore, the number of receptors for hexokinase II and glucose transporter type 4 (*GLUT4*) gene are increased. All these processes are conducting to an increase in glucose metabolism. On the other hand, it is able to phosphorylate and inhibit glycogen synthase, therefore leading to a diminution of glycogen manufacturing. AMPK also lowers liver gluconeogenesis by stimulating the phosphorylation of c-cyclic adenosine monophosphate-response element-binding protein (CREB-CBP) and dissociating the gluconeogenic CREB-CBP-target of rapamycin complex 2 (TORC2) transcriptional complex [173].

This happening is interceded by atypical protein kinase C (PKC *ι*/*λ*) and activates dismantling of the transcription and the suppression of the expression of the genes that control the gluconeogenesis enzymes. Moreover, metformin is able to activate AMPK, having therefore, favorable results on the lipid metabolism. Those effects are the decrease of cholesterol and fatty acid synthesis and are mediated through the inactivation of metformin-induced phosphorylation, 3-hydroxy-3-methylglutaryl (HMG)-CoA reductase, Acetyl-CoA carboxylase (ACC), the decrease in expression of the fatty acid synthase (FAS), and activation of malonyl-CoA carboxylase. Moreover, AMPK is able to inhibit SREBP-1c, which is involved in the synthesis of fatty acids [174].

Six open-label studies evaluated changes in liver histology, together with the levels of serum aminotransferase and improved markers of insulin resistance in patients with NAFLD that used metformin 1.2–2.0 g/day for 24 to 48 weeks as single treatment or combined with other drugs [172,175,176,177,178,179].

All of these studies reported an amelioration regarding insulin resistance: five of them reported a decrease of hepatic functional test rates and another one outlined a non-significant rise of them [175]. 

Concerning histopathological recovery, three researches revealed important variations after treatment with metformin regarding inflammation, steatosis, and fibrosis [172,176,177].

Contrasting to these outcomes, some lately open trials have discovered no advantages regarding the therapy with 1.5–1.7 g/day of metformin for 6–12 months. These studies followed its effects on insulin resistance, hepatic fatty changes and transaminases levels compared to lifestyle management or to the untreated lot. However, those studies had small samples of subjects. Moreover, these results might be due to the different doses of treatment and duration or the period of time within the biopsies [180,181,182].

Regarding metformin improvement on lipoprotein profiles, several authors have shown that this is able to lower the amounts of LDL cholesterol and triglycerides and to rise the amounts of high-density lipoprotein cholesterol [183].

In addition, metformin is able to reduce inflammatory markers, to improve hypercoagulation status and to increase fibrinolysis by lowering plasminogen activator inhibitor-1 levels and rising the action of tissue plasminogen activator. Moreover, metformin has the capacity to improve endothelial reactivity and the progression of atherosclerosis of the coronary arteries [184].

One other important process that is responsible for metformin’s capacity to improve endothelial dysfunction is the reduction of the oxidative components, such as the circulating advanced glycated end products (AGEs). In addition, it is capable of stimulating the intracellular AMPK, which leads to the activation of the endothelial isoform of NOSs [185].

#### 8.2.3. Newer Antihyperglycemic Agents

Concerning the newer antihyperglycemic agents, only glucagon-like peptide-1 receptor agonists (GLP-1 RAs) and sodium-glucose co-transporter-2 (SGLT-2) inhibitors seem to be useful in patients with NAFLD or NASH. GLP-1 RAs diminish the serum levels of hepatic enzymes and ameliorate fatty liver disease, as determined by liver biopsy or imaging methods [186]. Moreover, GLP-1 RAs decrease the chance of developing cardiovascular disease and kidney damage in subjects with T2DM [187]. In parallel, GLP-1 receptor agonists are able to reduce the epicardial adipose tissue and produce beneficial electrophysiological modifications in the atrium [188].

The mechanisms responsible for the NAFLD development are as follows: the accumulation of fat from increased FFAs uptake and lipogenesis and diminished elimination of the lipids from reduced fatty acid oxidation. Moreover, unhealthy lifestyle increases the levels of FFAs to the liver and upregulates de novo lipogenesis. In addition, insulin resistance in overweight subjects is responsible for lipolysis, therefore providing the flux of FFAs to the liver [189,190].

Regarding the occurrence of hepatic insulin resistance and NASH, the inflammatory cytokines play an important part. The source of these cytokines is the adipose tissue is insulin resistance [191]. Adiponectin is, as well, involved in the modulation of insulin resistance [192].

As it was discussed before, subjects with NAFLD have hypoadiponectinemia and adiponectin is associated with insulin sensitivity. It promotes the use of glucose and the β-oxidation of fatty acids, suppressing at the same time the fatty acid synthesis [193,194,195]. Therefore, NAFLD is strongly linked to hepatic, fat tissue and systemic insulin resistance [196].

GLP-1 receptor agonists: GLP-1 is produced by intestinal L cells and is secreted into the hepatic portal system. It is responsible for the stimulation of insulin by glucose-dependent mechanisms. In addition, GLP-1 is responsible for weight loss by reducing the secretion of glucagon, delaying gastric emptying and suppressing appetite. Preclinical studies have demonstrated that GLP-1 agonists are efficient in improving hepatic insulin sensitivity, steatosis and histology [197]. GLP-1 is responsible for improving signal transduction in adipocytes. This process is possible by its ability to upregulate the phosphorylation of Akt and expression of the protein of cyclins A, D1, and E [198]. The outcome of GLP-1 RAs on hepatic cells was studied in vitro. Exenatide was able to activate the genes that are associated to the oxidation of hepatic fatty acids and insulin sensitivity. These processes were observed in isolated hepatocytes from NASH rats. However, data is still needed in order to evaluate the presence of GLP-1 receptors in human hepatocytes [199,200]. Recent research regarding GLP-1 RAs effects on NAFLD reported that exenatide improved hepatic enzymes [201,202].

Many randomized controlled studies that used GLP-1 RAs for treating T2DM confirmed these findings. Moreover, the deduction on GLP-1 RAs effects on elevated aminotransferases was like a meta-analysis known as the ‘Liraglutide Effect and Action in Diabetes’ (LEAD) program [203]. 

Another GLP-1 RAs, lixisenatide was investigated in a meta-analysis that included 15 randomized controlled trials on T2DM subjects. They concluded that lixisenatide was able to increase the amount of overweight or obese patients who reach normal ALT distributions [204]. In addition, the effectiveness and safety of GLP-1 RAs were analyzed in a meta-analysis that indicated that this class of drugs is able to lower the levels of transaminases and ameliorate liver histology [205]. 

**Figure 2:**
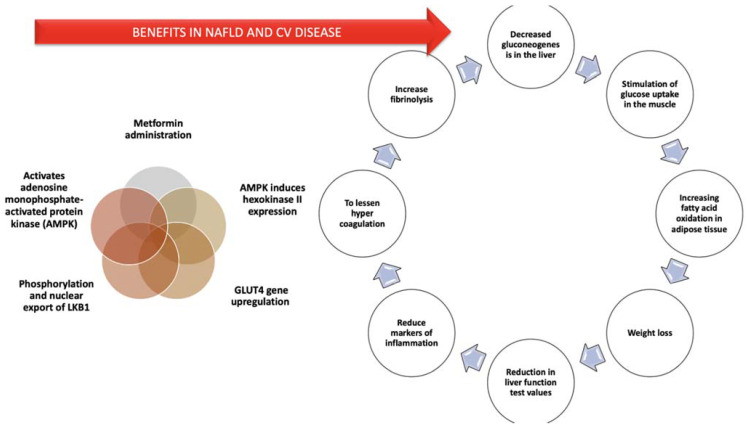
Benefits of metformin administration. Legend: AMPK: adenosine monophosphate-activated protein kinase; LKB1: liver kinase B1; GLUT4: Glucose transporter type 4.

A systematic review investigated GLP-1 RAs’ role in the treatment of NAFLD. Twenty-four clinical trials, 14 randomized control trials, and 10 different types of studies were taken into consideration. Six thousand three hundred and thirteen individuals were included in these studies. There was important heterogeneity in the quality of the study design, size of the sample, duration, placebo choice, and outcome measures. These data provided confirmation that GLP-1 RAs are able to improve the inflammation and fat accumulation in the liver. On the other hand, concerning GLP-1 RAs’ ability to improve the levels of fibrosis and to intercept with steatosis evolution to NASH and cirrhosis, prospective randomized control trials are still needed [206]. Figure 2 summarizes the main benefits of metformin administration. Studies have demonstrated for a long time that T2DM is associated with NAFLD, and serum transaminases levels are above the normal range, with predominating ALT levels [207].

Analyses carried out on animals have proven that SGLT-2 inhibitors are able to diminish the levels of transaminases, as well as hepatic steatosis and liver weight [208]. Several mechanisms are involved in the improvement of transaminases in subjects treated with SGLT-2 inhibitors. This medication is able to increase glucagon secretion from pancreatic α cells, therefore stimulating glucogenesis and β-oxidation of fatty acids in the liver. This process is accomplished by stimulating peroxisome proliferator-activated receptor α and carnitine palmitoyl transferase-1 [209,210].

Moreover, beside their capacity to reduce hepatic fat, SGLT-2 inhibitors can also reduce the inflammatory cytokine expression and collagen deposition in the liver [211]. Their role regarding the decrease of liver enzymes is due to the improvement of glycemic parameters and insulin resistance. A systemic review on 8 studies showed that 7 of them concluded that serum ALT and AST levels are reduced by using SGLT-2 inhibitors.

Shibuya et al. noticed a decline of ALT levels that has almost reached statistical significance, but information concerning AST levels was not available [210].

Six studies out of seven demonstrated a significantly decline of GGT levels, while another one, carried by Seko et al. found out that modification in serum GGT level almost reached statistical significance [212].

Although transaminases are indicators of hepatic histological modifications, a decrease of serum liver enzymes is not always associated with an improvement in liver histology [210].

Five researchers assessed the modifications regarding liver steatosis and revealed a reduction in patients using SGLT-2 inhibitors. However, no connection between modifications of ALT levels and changes regarding liver fat was found in the studies carried out by Shibuya et al. A link was although found in Sumida’s et al. research [210,213].

SGLT-2 inhibitor was examined in comparison to pioglitazone, a drug that was approved for the treatment of NAFLD. The decrease of hepatic fat was comparable in both drugs, despite the presence of diabetes. However, a study carried by Eriksson et al. showed that even though steatosis decreased with dapagliflozin treatment, it did not reach statistical significance compared to placebo. One reason why this study brought up this difference might be its lesser duration (12 weeks) [214].

Regarding the evolution of NAFLD to cirrhosis this is dictated by liver histology. Prospective studies that followed-up subjects for 20 years have shown that the possibility to progress to cirrhosis for NASH with fibrosis, NASH and simple steatosis are 38%, 25%, and 0–4% [215]. 

Fibrosis-4 (FIB-4) index is used as a non-invasive instrument for assessing hepatic fibrosis [216]. The parameters used to calculate it are as follows: the age, the number of thrombocytes, and transaminases levels. 

Two out of three studies found out that this index was decreased when using SGTL-2 inhibitors therapy. One of these studies carried out by Sumida et al. has also used in addition to the FIB-4 index the NAFLD fibrosis score [213].

NAFLD fibrosis score uses six variables: age, body mass index, hyperglycemia, thrombocytes count, albumin, and AST/ALT ratio. No remarkable modification in either indices was found in this research [217].

Weight loss concerning mainly fat mass loss while being under the treatment with SGLT-2 inhibitors is associated with glycosuria. Previous meta-analysis showed that weight loss of more than 5% was associated with improved hepatic steatosis and loss of more than 7% improved the histological characteristics of NAFLD. Therefore, SGLT-2 inhibitors’ ability to improve insulin resistance and weight may ameliorate liver steatosis. The improvement of insulin resistance by SGLT-2 inhibitors is able to decrease the apoptosis and inflammation of hepatocytes [218,219].

Studies that used rat/mice model with NAFLD/NASH and diabetes found out that the use of SGLT-2 inhibitors was able to decrease de novo lipogenesis and increase fatty acid β-oxidation, therefore improving hepatic steatosis [209].

As it was discussed before, SGLT-2 inhibitors are capable of decreasing glycaemia levels and ameliorate insulin resistance by promoting glycosuria. This control of the glycaemia is able to downregulate carbohydrate responsive element-binding protein. The latter mentioned is a transcription element in charge of the activation of fatty acid synthesis. In addition, the improvement of insulin resistance downregulates sterol regulatory element binding-protein 1c, which is also a transcription element connected to lipid manufacturing and inhibition of de novo lipogenesis [220].

Moreover, SGLT-2 inhibitors appear to have favorable consequences on NAFLD/NASH throughout mechanisms that are independent of weight and glucose, as it was shown in several studies. Those mechanisms involve the reduction of inflammatory markers, oxidative stress, and lipogenesis and improvement of FFAs oxidation [221].

Concerning SGLT-2 inhibitors’ role on the glucagon-to-insulin ratio, they are able to improve it, therefore increasing ketogenesis in the liver and leading to protection against steatosis [222].

In one study, carried out by Akuta et al., canagliflozin, an SGLT-2 inhibitor was used for a long term to treat seven patients diagnosed with NAFLD and T2DM. Liver biopsies were performed pretreatment and at 24 weeks and one year following the beginning of therapy. Six of the subjects were also assessed by biopsy after three or more years.

At the third hepatic biopsy all patients worsened their body mass index and waist circumference. Yet, the count of hepatic fat accumulation, lobular inflammation, ballooning, and fibrosis ameliorated on the third liver biopsy. However, one patient had histopathological worsening at the third liver biopsy. As a conclusion, long-term therapy of NAFLD complicated by T2DM utilizing SGLT-2 inhibitors is able to improve the liver histopathology although clinical features might be worsened [223].

### 8.3. Hypertension Treatment 

The powerful relationship and almost same pathogenic description of those two entities propose that antihypertensive therapy could also be useful in NAFLD patients [224].

#### 8.3.1. Renin-Angiotensin-Aldosterone System Inhibitors 

Studies have shown that this class of drugs has promising results in this pathology. Kaji et al. combined losartan, an angiotensin II type I receptor blocker, with deferasirox, an oral iron chelator in rats with NASH. Their results indicated that this dual combination attenuated the progression of NASH [225]. Moreover, sartans lead to the amelioration of insulin resistance and changes in liver fibrosis [226].

In addition, telmisartan seems to be able to decrease the levels of serum free fatty acid and steatosis [227].

#### 8.3.2. Probiotics and Prebiotics 

Gut microbiome alteration might be a cause for the development of NAFLD and hypertension. There are studies that suggest that the use of pro- and prebiotics might be an alternative therapy for NAFLD and that probiotics could induce a decrease in blood pressure [52].

### 8.4. Other Drugs That Might Have a Benefit in Non-Alcoholic Fatty Liver Disease

Farnesoid X receptor agonists: farnesoid X receptor is a regulator of lipid and glucose metabolism and inflammatory and fibrotic processes. An international phase III clinical trial of obeticholic acid, a synthetic ligand of farnesoid X receptor, in NASH patients is now ongoing and is evaluating obeticholic acid compared to placebo on liver biochemistry and markers of liver function [228].

Fibroblast Growth Factor 21 Analogs (*FGF21*) is primarily secreted by the liver and adipose tissue and has multiple effects on lipid and glucose metabolism and insulin sensitivity. It is considered to protect against NAFLD [13]. A phase 2a trial with Pegbelfermin, a polyethylene glycolated (PEGylated) fibroblast growth factor 21 analogue has demonstrated that its administration for 16 weeks has notably reduced hepatic fat in NASH patients, and it was well tolerated [229].

CCR2 (C-C motif chemokine receptor 2) and CCR5 Antagonists. CCR are chemokine receptors involved in the pathophysiology of NAFLD through their promotion of local macrophage infiltration and fibrogenesis [19]. In the phase IIb CENTAUR (Efficacy and Safety Study of Cenicriviroc for the Treatment of NASH in Adult Participants With Liver Fibrosis) trial, treatment with cenicriviroc significantly improved fibrosis with no aggravation of steatohepatitis in comparison to placebo treatment [230]. 

## 9. Conclusions

NAFLD is a condition with a growing prevalence. Cardiovascular disease appears to be strongly connected with liver steatosis and is the major cause of deaths despite NAFLD’s natural evolution to cirrhosis and hepatocarcinoma. Recent years have raised interest regarding its pathophysiology and possible therapeutic management. As NAFLD is shown to be linked to metabolic syndrome, controlling the risk factors should be the primary prevention of fatty liver disease and its associated comorbidities. Multidisciplinary approach of these patients in order to control related risk factors and to evaluate for cardiovascular and hepatic complications should be done. To better understand the possible mechanisms and, therefore, therapeutic management that derives from them, further interventional studies regarding the association between NAFLD and cardiovascular disease are needed.

## References

[1] Jonathan C.C., Jay D.H., Helen H.H. (2011). Human Fatty Liver Disease: Old Questions and New Insights. Science.

[2] Younossi Z.M., Koenig A.B., Abdelatif D., Fazel Y., Henry L., Wymer M. (2016). Global Epidemiology of Nonalcoholic Fatty Liver Disease-Meta-Analytic Assessment of Prevalence, Incidence, and Outcomes. Hepatology.

[3] Lindenmeyer C.C., McCullough A.J. (2018). The Natural History of Nonalcoholic Fatty Liver Disease—An Evolving View. Clin. Liver. Dis..

[4] Zhu J.Z., Hollis-Hansen K., Wan X.Y., Fei S.J., Pang X.L., Meng F.D., Yu C.H., Li Y.M. (2016). Clinical Guidelines of Non-Alcoholic Fatty Liver Disease: A Systematic Review. World J. Gastroenterol..

[5] Saverymuttu S.H., Joseph A.E., Maxwell J.D. (1986). Ultrasound Scanning in the Detection of Hepatic Fibrosis and Steatosis. Br. Med. J. Clin. Res..

[6] Thomas E.L., Hamilton G., Patel N., O’Dwyer R., Dore C.J., Goldin R.D., Bell J.D., Taylor-Robinson S.D. (2005). Hepatic Triglyceride Content and Its Relation to Body Adiposity: A Magnetic Resonance Imaging and Proton Magnetic Resonance Spectroscopy Study. Gut.

[7] Tang A., Desai A., Hamilton G., Wolfson T., Gamst A., Lam J., Clark L., Hooker J., Chavez T., Ang B.D. (2015). Accuracy of MR Imaging Estimated Proton Density Fat Fraction for Classification of Dichotomized Histologic Steatosis Grades in Nonalcoholic Fatty Liver Disease. Radiology.

[8] Yoshio S., Atsushi N., Yoshito I. (2014). Limitations of Liver Biopsy and Non-Invasive Diagnostic Tests for the Diagnosis of Nonalcoholic Fatty Liver Disease/Nonalcoholic Steatohepatitis. World J. Gastroenterol..

[9] Brunt E.M., Janney C.G., Di Bisceglie A.M., Neuschwander-Tetri B.A., Bacon B.R. (1999). Nonalcoholic Steatohepatitis: A Proposal for Grading and Staging the Histological Lesions. Am. J. Gastroenterol..

[10] Neuman M.G., Malnick S., Maor Y., Nanau R.M., Melzer E., Ferenci P., Seitz H.K., Mueller S., Mell H., Samuel D. (2015). Alcoholic Liver Disease: Clinical and Translational Research. Exp. Mol. Pathol..

[11] Park S.H., Jeon W.K., Kim S.H., Kim H.J., Park D.I., Cho Y.K., Sung I.K., Sohn C.I., Keum D.K., Kim B.I. (2006). Prevalence and Risk Factors of Non-Alcoholic Fatty Liver Disease among Korean Adults. J. Gastroenterol. Hepatol..

[12] Anstee Q.M., Targher G., Day C.P. (2013). Nat Rev Progression of NAFLD to Diabetes Mellitus, Cardiovascular Disease or Cirrhosis. Gastroenterol. Hepatol..

[13] Yan-Ci Z., Guo-Jun Z., Ze C., Zhi-Gang S., Jingjing C., Hongliang L. (2020). Nonalcoholic Fatty Liver Disease An Emerging Driver of Hypertension. Hypertension.

[14] Catena C., Bernardi S., Sabato N., Grillo A., Ermani M., Sechi L.A., Fabris B., Carretta R., Fallo F. (2012). Ambulatory Arterial Stiffness Indices and Nonalcoholic Fatty Liver Disease in Essential Hypertension. Nutr. Metab. Cardiovasc. Dis..

[15] Lin Y.C., Lo H.M., Chen J.D. (2005). Sonographic Fatty Liver, Overweight and Ischemic Heart Disease. World J. Gastroenterol..

[16] Schindhelm R.K., Dekker J.M., Nijpels G., Bouter L.M., Stehouwer C.D.A., Heine R.J., Diamant M. (2007). Alanine Aminotransferase Predicts Coronary Heart Disease Events: A 10-Year Follow-up of the Hoorn Study. Atherosclerosis.

[17] Sinn D.H., Gwak G.Y., Park H.N., Kim J.E., Min Y.W., Kim K.M., Kim Y.J., Choi M.S., Lee J.H., Koh K.C. (2012). Ultrasonographically Detected Non-Alcoholic Fatty Liver Disease Is an Independent Predictor for Identifying Patients with Insulin Resistance in Non-Obese, Non-Diabetic Middle-Aged Asian Adults. Am. J. Gastroenterol..

[18] Musso G., Gambino R., Bo S., Uberti S., Biroli G., Pagano G., Cassader M. (2008). Should Nonalcoholic Fatty Liver Disease Be Included in the Definition of Metabolic Syndrome? A Cross-Sectional Comparison with Adult Treatment Panel III Criteria in Nonobese Nondiabetic Subjects. Diabetes Care.

[19] Haukeland J.W., Damås J.K., Konopski Z., Løberg E.M., Haaland T., Goverud I., Torjesen P.A., Birkeland K., Bjøro K., Aukrust P. (2006). Systemic Inflammation in Nonalcoholic Fatty Liver Disease Is Characterized by Elevated Levels of CCL2. J. Hepatol..

[20] Stumpf C., Auer C., Yilmaz A., Lewczuk P., Klinghammer L., Schneider M., Daniel W.G., Schmieder R.E., Garlichs C.D. (2011). Serum Levels of the Th1 Chemoattractant Interferon-γ-inducible Protein (IP) 10 Are Elevated in Patients with Essential Hypertension. Hypertens. Res..

[21] Bai L., Li H.I. (2019). Immune Regulatory Networks in Hepatic Lipid Metabolism. J. Mol. Med. Berl..

[22] Meex R.C.R., Watt M.J. (2017). Hepatokines: Linking Nonalcoholic Fatty Liver Disease and Insulin Resistance. Nat. Rev. Endocrinol..

[23] Nunes K.P., de Oliveira A.A., Mowry F.E., Biancardi V.C. (2019). Targeting Toll-like Receptor 4 Signalling Pathways: Can Therapeutics Pay the Toll for Hypertension?. Br. J. Pharmacol..

[24] Sinn D.H., Kang D., Jang H.R., Gu S., Cho S.J., Paik S.W., Ryu S., Chang Y., Lazo M., Guallar E. (2017). Development of Chronic Kidney Disease in Patients with Non-Alcoholic Fatty Liver Disease: A Cohort Study. J. Hepatol..

[25] Artunc F., Schleicher E., Weigert C., Fritsche A., Stefan N., Häring H.U. (2016). The Impact of Insulin Resistance on the Kidney and Vasculature. Nat. Rev. Nephrol..

[26] Watt M.J., Miotto P.M., De Nardo W., Montgomery M.K. (2019). The Liver as an Endocrine Organ—Linking NAFLD and Insulin Resistance. Endocr. Rev..

[27] Landsberg L., Young J.B. (1985). Insulin Mediated Glucose Metabolism in the Relationship between Dietary Intake and Sympathetic Nervous System Activity. Int. J. Obes..

[28] Jiao N., Baker S.S., Chapa-Rodriguez A., Liu W., Nugent C.A., Tsompana M., Mastrandrea L., Buck M.J., Baker R.D., Genco R.J. (2018). Suppressed Hepatic Bile Acid Signalling despite Elevated Production of Primary and Secondary Bile Acids in NAFLD. Gut.

[29] Kasumov T., Edmison J.M., Dasarathy S., Bennett C., Lopez R., Kalhan S.C. (2011). Plasma levels of asymmetric dimethylarginine (ADMA) in patients with biopsy-proven non-alcoholic fatty liver disease. Metabolism.

[30] Serg M., Kampus P., Kals J., Zagura M., Muda P., Tuomainen T.P., Zilmer K., Salum E., Zilmer M., Eha J. (2011). Association between Asymmetric Dimethylarginine and Indices of Vascular Function in Patients with Essential Hypertension. Blood Press.

[31] Sydow K., Mondon C.E., Cooke J.P. (2005). Insulin Resistance: Potential Role of the Endogenous Nitric Oxide Synthase Inhibitor ADMA. Vasc. Med..

[32] Abbasi F., Asagmi T., Cooke J.P., Lamendola C., McLaughlin T., Reaven G.M., Stuehlinger M., Tsao P.S. (2001). Plasma Concentrations of Asymmetric Dimethylarginine Are Increased in Patients with Type 2 Diabetes Mellitus. Am. J. Cardiol..

[33] Villela-Nogueira C.A., Leite N.C., Cardoso C.R.L., Salles G.F. (2014). NAFLD and Increased Aortic Stiffness: Parallel or Common Physiopathological Mechanisms?. World J. Gastroenterol..

[34] Johnson C.P., Baugh R., Wilson C.A., Burns J. (2001). Age Related Changes in the Tunica Media of the Vertebral Artery: Implications for the Assessment of Vessels Injured by Trauma. J. Clin. Pathol..

[35] Nickenig G., Roling J., Strehlow K., Schnabel P., Bohm M. (1998). Insulin Induces Upregulation of Vascular AT1 Receptor Gene Expression by Posttranscriptional Mechanisms. Circulation.

[36] Jesmin S., Sakuma I., Salah-Eldin A., Nonomura K., Hattori Y., Kitabatake A. (2003). Diminished Penile Expression of Vascular Endothelial Growth Factor and Its Receptors at the Insulin-Resistant Stage of a Type II Diabetic Rat Model: A Possible Cause for Erectile Dysfunction in Diabetes. J. Mol. Endocrinol..

[37] Rizzoni D., Porteri E., Guelfi D., Muiesan M.L., Valentini U., Cimino A., Girelli A., Rodella L., Bianchi R., Sleiman I. (2001). Structural Alterations in Subcutaneous Small Arteries of Normotensive and Hypertensive Patients with Non-Insulin-Dependent Diabetes Mellitus. Circulation.

[38] Vlachopoulos C., Manesis E., Baou K., Papatheodoridis G., Koskinas J., Tiniakos D., Aznaouridis K., Archimandritis A., Stefanadis C. (2010). Increased Arterial Stiffness and Impaired Endothelial Function in Nonalcoholic Fatty Liver Disease: A Pilot Study. Am. J. Hypertens..

[39] Kim B.J., Kim N.H., Kim B.S., Kang J.H. (2012). The Association between Nonalcoholic Fatty Liver Disease, Metabolic Syndrome and Arterial Stiffness in Nondiabetic, Nonhypertensive Individuals. Cardiology.

[40] Huang Y., Bi Y., Xu M., Ma Z., Xu Y., Wang T., Li M., Liu Y., Lu J., Chen Y. (2012). Nonalcoholic Fatty Liver Disease Is Associated with Atherosclerosis in Middle-Aged and Elderly Chinese. Arter. Thromb. Vasc. Biol..

[41] Lee Y.J., Shim J.Y., Moon B.S., Shin Y.H., Jung D.H., Lee J.H., Lee H.R. (2012). The Relationship between Arterial Stiffness and Nonalcoholic Fatty Liver Disease. Dig. Sci..

[42] Ozturk A.K., Uygun A.A., Guler B.A.K., Demirci A.H., Ozdemir C.C., Cakir B.M., Sakin A.Y.S., Turker D.T., Sari E.S., Demirbas B.S. (2015). Nonalcoholic Fatty Liver Disease Is an Independent Risk Factor for Atherosclerosis in Young Adult Men. Atherosclerosis.

[43] Jain S., Khera R., Corrales-Medina V.F., Townsend R.R., Chirinos J.A. (2014). Inflammation and Arterial Stiffness in Humans. Atherosclerosis.

[44] Farzanegi P., Amir D., Ebrahimpoor Z., Mahdieh A., Azarbayjani M.A. (2019). Mechanisms of Beneficial Effects of Exercise Training on Non-Alcoholic Fatty Liver Disease (NAFLD): Roles of Oxidative Stress and Inflammation. Eur. J. Sport Sci..

[45] Colagar A.H., Marzony E.T. (2009). Ascorbic Acid in Human Seminal Plasma: Determination and Its Relationship to Sperm Quality. J. Clin. Biochem. Nutr..

[46] Yu Y., Cai J., She Z., Li H. (2019). Insights into the Epidemiology, Pathogenesis, and Therapeutics of Nonalcoholic Fatty Liver Diseases. Adv. Sci. Weinh..

[47] Distrutti E., Mencarelli A., Santucci L., Renga B., Orlandi S., Donini A., Shah V., Fiorucci S. (2008). The Methionine Connection: Homocysteine and Hydrogen Sulfide Exert Opposite Effects on Hepatic Microcirculation in Rats. Hepatology.

[48] Polimeni L., Del Ben M., Baratta F., Perri L., Albanese F., Pastori D., Violi F., Angelico F. (2015). Oxidative Stress: New Insights on the Association of Non-Alcoholic Fatty Liver Disease and Atherosclerosis. World J. Hepatol..

[49] Wang Y., Chen S., Yao T., Li D., Wang Y., Li Y., Wu S., Cai J. (2014). Homocysteine; as a Risk Factor for Hypertension: A 2-Year Follow-up Study. PLoS ONE.

[50] Björkholm B., Bok C.M., Lundin A., Rafter J., Hibberd M.L., Pettersson S. (2009). Intestinal Microbiota Regulate Xenobiotic Metabolism in the Liver. PLoS ONE.

[51] Safari Z., Gérard P. (2019). The Links between the Gut Microbiome and Non Alcoholic Fatty Liver Disease (NAFLD). Cell. Mol. Life Sci..

[52] Marques F.Z., Mackay C.R., Kaye D.M. (2018). Beyond Gut Feelings; How the Gut Microbiota Regulates Blood Pressure. Nat. Rev. Cardiol..

[53] Del Campo J.A., Gallego-Durán R., Gallego P., Grande L. (2018). Genetic and Epigenetic Regulation in Nonalcoholic Fatty Liver Disease (NAFLD). Int. J. Mol. Sci..

[54] Fan W., Qu X., Li J., Wang X., Bai Y., Cao Q., Ma L., Zhou X., Zhu A.W., Liu B.W. (2017). Associations between Polymorphisms of the ADIPOQ Gene and Hypertension Risk: A Systematic and Meta-Analysis. Sci. Rep..

[55] Zhu W., Cheng K.K., Vanhoutte P.M., Lam K.S., Xu A. (2008). Vascular Effects of Adiponectin: Molecular Mechanisms and Potential Therapeutic Intervention. Clin. Sci..

[56] Musso G., Saba F., Cassader M., Paschetta E., De Michieli F., Pinach S., Framarin L., Berrutti M., Leone N., Parente R. (2019). Angiotensin II Type 1 Receptor Rs5186 Gene Variant Predicts Incident NAFLD and Associated Hypertension: Role of Dietary Fat-Induced pro-Inflammatory Cell Activation. Am. J. Gastroenterol..

[57] Srivastava R.A.K. (2018). Life-Style-Induced Metabolic Derangement and Epigenetic Changes Promote Diabetes and Oxidative Stress Leading to NASH and Atherosclerosis Severity. J. Diabetes Metab. Disord..

[58] Costantino S., Mohammed S.A., Ambrosini S., Paneni F. (2019). Epigenetic Processing in Cardiometabolic Disease. Atherosclerosis.

[59] Bungau S., Behl T., Tit D.M., Banica F., Bratu O.G., Diaconu C.C., Nistor-Cseppento C.D., Bustea C., Aron R.A.C., Vesa C.M. (2020). Interactions between Leptin and Insulin Resistance in Patients with Prediabetes, with and without NAFLD. Exp. Ther. Med..

[60] Gheorghe G., Bungau S., Ceobanu G., Ilie M., Bacalbasa N., Bratu O.G., Vesa C.M., Gaman M.A., Diaconu C.C. (2021). The Non-Invasive Assessment of Hepatic Fibrosis. J. Formos. Med. Assoc..

[61] Ampuero J., Gallego-Durán R., Romero-Gómez M. (2015). Association of NAFLD with Subclinical Atherosclerosis and Coronary-Artery Disease: Meta-Analysis. J. Rev. Esp. Enferm. Dig..

[62] Yoosoo C., Seungho R., Ki-Chul S., Yong K.C., Eunju S., Han-Na K., Hyun-Suk J., Kyung E.Y., Jiin A., Hocheol S. (2019). Alcoholic and Non-Alcoholic Fatty Liver Disease and Associations with Coronary Artery Calcification (CAC): Evidence from the Kangbuk Samsung Health Study. Gut.

[63] Treeprasertsuk S., Lopez-Jimenez F., Lindor K.D. (2011). Nonalcoholic Fatty Liver Disease and the Coronary Artery Disease. Dig. Sci..

[64] Kotronen A., Juurinen L., Tiikkainen M., Vehkavaara S., Järvinen Hannele Y. (2008). Increased Liver Fat, Impaired Insulin Clearance, and Hepatic and Adipose Tissue Insulin Resistance in Type 2 Diabetes. Gastroenterology.

[65] Tarantino G., Caputi A. (2011). JNKs, Insulin Resistance and Inflammation: A Possible Link between NAFLD and Coronary Artery Disease. World J. Gastroenterol..

[66] Ebato C., Uchida T., Arakawa M., Komatsu M., Ueno T., Komiya K., Azuma K., Hirose T., Tanaka K., Kominami E. (2008). Autophagy Is Important in Islet Homeostasis and Compensatory Increase of b Cell Mass in Response to High-Fat Diet. Cell. Metab..

[67] Weickert M.O. (2006). Signalling Mechanisms Linking Hepatic Glucose and Lipid Metabolism. Pfeiffer. AF Diabetol..

[68] Nguyen M.T., Satoh H., Favelyukis S., Babendure J.L., Imamura T., Sbodio J.I., Zalevsky J., Dahiyat B.I., Chi N.W., Olefsky J.M. (2005). JNK and Tumor Necrosis Factor-Alpha Mediate Free Fatty Acid-Induced Insulin Resistance in 3T3-L1 Adipocytes. J. Biol. Chem..

[69] Yilmaz Y., Kurt R., Gurdal A., Alahdaba Y.O., Yonal O., Senates E., Polat N., Erend F., Imeryuz N., Oflaz H. (2011). Circulating Vaspin Levels and Epicardial Adipose Tissue Thickness Are Associated with Impaired Coronary Flow Reserve in Patients with Nonalcoholic Fatty Liver Disease. Atherosclerosis.

[70] Crespo J., Cayon A., Fernandez-Gil P., Hernandez-Guerra M., Mayorga M., Dominguez-Diez A., Fernandez -Escalante J.C., Pons-Romero F. (2001). Gene Expression of Tumor Necrosis Factor and TNF-Receptors, P55 and P75, in Nonalcoholic Steatohepatitis Patients. Hepatology.

[71] Maeda N., Shimomura I., Kishida K., Nishizawa H., Matsuda M., Nagaretani H., Furuyama N., Kondo H., Takahashi M., Arita Y. (2002). Diet-Induced Insulin Resistance in Mice Lacking Adiponectin/ACRP30. Nat. Med..

[72] Xu A., Wang Y., Keshaw H., Xu L.Y., Lam K.S.L., Cooper G.J.S. (2003). The Fat-Derived Hormone Adiponectin Alleviates Alcoholic and Nonalcoholic Fatty Liver Diseases in Mice. J. Clin. Investig..

[73] McKimmie R.L., Daniel K.R., Carr J.J., Bowden D.W., Freedman B.I., Register T.C., Hsu F.C., Lohman K.K., Weinberg R.B., Wagenknecht L.E. (2008). Hepatic Steatosis and Subclinical Cardiovascular Disease in a Cohort Enriched for Type 2 Diabetes: The Diabetes Heart Study. Am. J. Gastroenterol..

[74] Otsuka F., Sugiyama S., Kojima S., Maruyoshi H., Funahashi T., Sakamoto T., Yoshimura K., Kimura K., Umemura S., Ogawa H. (2007). Hypoadiponectinemia Is Associated with Impaired Glucose Tolerance and Coronary Artery Disease in Non-Diabetic Men. Circ. J..

[75] Edmison J., McCullough A.J. (2007). Pathogenesis of Non-Alcoholic Steatohepatitis: Human Data. Clin. Liver Dis..

[76] Solga S.F., Diehl A.M. (2003). Nonalcoholic Fatty Liver Disease: Lumen-Liver Interactions and Possible Role for Probiotics. J. Hepatol..

[77] Wanless I.R., Bargman J.M., Oreopoullos D.G., Vas S.I. (1989). Subcapsular Steatonecrosis in Response to Peritoneal Insulin Deliver: A Clue to the Pathogenesis of Steatonecrosis in Obesity. Mod. Pathol..

[78] Khalili K., Lan F.P., Hanbidge A.E., Muradali D., Oreopoulos D.G., Wanless I.R. (2003). Hepatic Subcapsular Steatosis in Response to Intraperitoneal Insulin Delivery: CT Findings and Prevalence. AJR Am. J. Roentgenol..

[79] Goldstein B.J., Kalyankar M., Wu X. (2005). Insulin Action Is Facilitated by Insulin-Stimulated Reactive Oxygen Species with Multiple Potential Signaling Targets. Diabetes.

[80] Li X.L., Man K., Ng K.T., Lee T.K., Lo C.M., Fan S.T. (2004). Insulin in UW Solution Exacerbates Hepatic Ischemia/Reperfusion Injury by Energy Depletion through the IRS-2/SREBP-1c Pathway. Liver Transpl..

[81] Ozcan U., Cao Q., Yilmaz E., Lee A.H., Iwakoshi N.N., Ozdelen E., Tuncman G., Gorgun C., Glimcher L.H., Hotamisligil G.S. (2004). Endoplasmic Reticulum Stress Links Obesity, Insulin Action, and Type 2 Diabetes. Science.

[82] Fargion S., Mattioli M., Fracanzani A.L., Sampietro M., Tavazzi D., Fociani P., Taioli E., Valenti L., Fiorelli G. (2001). Hyperferritinemia, Iron Overload, and Multiple Metabolic Alterations Identify Patients at Risk for Nonalcoholic Steatohepatitis. Am. J. Gastroenterol..

[83] Fernandez Real J.M., Casamitjana-Abella R., Ricart-Engel W., Arroyo E., Balanca R., Casamitjana-Abella R., Cabrero D., Fernandez-Castaner M., Soler J. (1998). Serum Ferritin as a Component of the Insulin Resistance Syndrome. Diabetes Care.

[84] Mendler M.-H., Turlin B., Moirand R., Jouanolle A.-M., Sapey T., Guyader D., le Gall J.-Y., Brissot P., David V., Deugnier Y. (1999). Insulin Resistance-Associated Hepatic Iron Overload. Gastroenterology.

[85] Riquelme A., Soza A., Nazal L., Martinez G., Kolbach M., Patillo A., Arellano M., Duarte I., Martinez J., Molgo M. (2004). Histological Resolution of Steatohepatitis after Iron Depletion. Dig. Sci..

[86] Clarke S.D. (2001). Nonalcoholic Steatosis and Steatohepatitis. I. Molecular Mechanism for Polyunsaturated Fatty Acid Regulation of Gene Transcription. Am. J. Physiol. Gastrointest. Liver Physiol..

[87] Tafani M., Schneider T.G., Pastorino J.G., Farber J.L. (2000). Cytochrome Dependent Activation of Caspase 3 by Tumor Necrosis Factor Requires Induction of the Mitochondrial Permeability Transition. Am. J. Pathol..

[88] Pastorini J.G., Simbula G., Yamamoto K., Glascott P.A., Rothman R.J., Farber J.L. (1996). Cytotoxicity of TNF Depends on Induction of the Mitochondrial Permeability Transition. J. Biochem..

[89] Paradis V., Perle G., Bonvoust F., Dargere D., Parfait B., Vidaud M., Conti M., Huet S., Ba N., Buffet C. (2001). High Glucose and Hyperinsulinemia Stimulate Connective Tissue Growth Factor Expression: A Potential Mechanism Involved in Progression to Fibrosis in Nonalcoholic Steatohepatitis. Hepatology.

[90] Poli G. (2000). Pathogenesis of Liver Fibrosis: The Role of Oxidative Stress. Mol Asp. Med..

[91] Canbay A., Taimr P., Torok N., Higuchi H., Friedman S., Gores G.J. (2003). Apoptotic Body Engulfment by a Human Stellate Cell Line Is Profibrogenic. Lab. Investig..

[92] Feldstein A.E., Canbay A., Angulo P., Taniai M., Burgart L.J., Lindor K.D., Gores G.J. (2003). Hepatocyte Apoptosis and FAS Expression Are Prominent Features of Human Nonalcoholic Steatohepatitis. Gastroenterology.

[93] Li Z., Oben J.A., Yang S., Lin H., Stafford E.A., Soloski M.J., Thomas S.A., Diehl A.M. (2004). Norepinephrine Regulates Hepatic Innate Immune System in Leptin Deficient Mice with Nonalcoholic Steatohepatitis. Hepatology.

[94] Käräjämäki A.J., Olli-Pekka P., Markku S., Kesäniemi Y.A., Heikki H., Ukkola O. (2015). Non-Alcoholic Fatty Liver Disease as a Predictor of Atrial Fibrillation in Middle-Aged Population (OPERA Study). PLoS ONE.

[95] Targher G., Valbuso F., Bonapace S., Bertolini L., Zenari L., Rodella S., Zoppini G., Mantovani W., Barbieri E., Byrne C.D. (2013). Non-Alcoholic Fatty Liver Disease Is Associated with an Increased Incidence of Atrial Fibrillation in Patients with Type 2 Diabetes. Clin. Sci..

[96] Hung C.S., Tseng P.H., Tu C.H., Chen C.C., Liao W.C., Lee Y.C., Chiu H.M., Lin H.J., Ho Y.L., Yang W.S. (2015). Nonalcoholic Fatty Liver Disease Is Associated with QT Prolongation in the General Population. J. Am. Heart Assoc..

[97] Mantovani A., Rigamonti A., Bonapace S., Bolzan B., Pernigo M., Morani F., Giovanni L., Bergamini C., Bertolini L., Valbusa F. (2016). Nonalcoholic Fatty Liver Disease Is Associated with Ventricular Arrhythmias in Patients with Type 2 Diabetes Referred for Clinically Indicated 24-h Holter Monitoring. Diabetes Care.

[98] Mantovani A., Rigolon R., Bonapace S., Morani G., Zoppini G., Bonora E., Targher G. (2017). Nonalcoholic Fatty Liver Disease Is Associated with an Increased Risk of Heart Block in Hospitalized Patients with Type 2 Diabetes Mellitus. PLoS ONE.

[99] Tsang T.S., Barnes M.E., Miyasaka Y., Cha S.S., Bailey K.R., Verzosa G.C., Seward J.B., Gersh B.J. (2008). Obesity as a Risk Factor for the Progression of Paroxysmal to Permanent Atrial Fibrillation: A Longitudinal Cohort Study of 21 Years. Eur. Heart J..

[100] European Association for the Study of the Liver (EASL), European Association for the Study of Diabetes (EASD), European Association for the Study of Obesity (EASO) (2016). EASL-EASD-EASO Clinical Practice Guidelines for the Management of Non-Alcoholic Fatty Liver Disease. Diabetologia.

[101] Graner M., Nyman K., Siren R., Pentikainen M.O., Lundbom J., Hakkarainen A., Lauerma K., Lundbom N., Nieminen M.S., Taskinen M.R. (2014). Ectopic Fat Depots and Left Ventricular Function in Nondiabetic Men with Nonalcoholic Fatty Liver Disease. Circ. Cardiovasc. Imaging.

[102] Käräjämäki A.J., Hukkanen J., Ukkola O. (2018). The Association of Non-Alcoholic Fatty Liver Disease and Atrial Fibrillation: A Review. Ann. Med..

[103] Liu Y.C., Hung C.S., Wu Y.W., Lee Y.C., Lin Y.H., Lin C., Lo M.T., Chan C.C., Ma H.P., Ho Y.L. (2013). Influence of Non-Alcoholic Fatty Liver Disease on Autonomic Changes Evaluated by the Time Domain, Frequency Domain, and Symbolic Dynamics of Heart Rate Variability. PLoS ONE.

[104] Park H.W., Shen M.J., Lin S.F., Fishbein M.C., Chen L.S., Chen P.S. (2012). Neural Mechanisms of Atrial Fibrillation. Curr. Opin. Cardiol..

[105] Guo Y., Lip G.Y., Apostolakis S. (2012). Inflammation in Atrial Fibrillation. J. Am. Coll. Cardiol..

[106] Ding Y.H., Ma Y., Qian L.Y., Xu Q., Wang L.H., Huang D.S., Zou H. (2017). Linking Atrial Fibrillation with Non-Alcoholic Fatty Liver Disease: Potential Common Therapeutic Targets. Oncotarget.

[107] Lin Y.K., Chen Y.C., Chen J.H., Chen S.A., Chen Y.J. (2012). Adipocytes Modulate the Electrophysiology of Atrial Myocytes: Implications in Obesity-Induced Atrial Fibrillation. Basic Res. Cardiol..

[108] Chung M.K., Martin D.O., Sprecher D., Wazni O., Kanderian A., Carnes C.A., Bauer J.A., Tchou P.J., Niebauer M.J., Natale A. (2001). C-Reactive Protein Elevation in Patients with Atrial Arrhythmias: Inflammatory Mechanisms and Persistence of Atrial Fibrillation. Circulation.

[109] Mantovani A., Pernigo M., Bergamini C., Bonapace S., Lipari P., Valbusa F., Bertolini L., Zenari L., Pichiri I., Dauriz M. (2015). Heart Valve Calcification in Patients with Type 2 Diabetes and Nonalcoholic Fatty Liver Disease. Metabolism.

[110] Mantovani A. (2017). Nonalcoholic Fatty Liver Disease (NAFLD) and Risk of Cardiac Arrhythmias: A New Aspect of the Liver-Heart Axis. J. Clin. Transl. Hepatol..

[111] Ballestri S., Lonardo A., Bonapace S., Byrne C.D., Loria P., Targher G. (2014). Risk of Cardiovascular, Cardiac and Arrhythmic Complications in Patients with Non-Alcoholic Fatty Liver Disease. World J. Gastroenterol..

[112] Perseghin G., Lattuada G., De Cobelli F., Esposito A., Belloni E., Ntali G., Ragogna F., Canu T., Scifo P., Del Maschio A. (2008). Increased Mediastinal Fat and Impaired Left Ventricular Energy Metabolism in Young Men with Newly Found Fatty Liver. Hepatology.

[113] Camici P., Ferrannini E., Opie L.H. (1989). Myocardial Metabolism in Ischemic Heart Disease: Basic Principles and Application to Imaging by Positron Emission Tomography. Prog. Cardiovasc. Dis..

[114] Taegtmeyer H., McNulty P., Young M.E. (2002). Adaptation and Maladaptation of the Heart in Diabetes: Part I: General Concepts. Circulation.

[115] Alp H., Karaarslan S., Eklioğlu B.S., Atabek M.E., Altın H., Baysal T. (2013). Association between Nonalcoholic Fatty Liver Disease and Cardiovascular Risk in Obese Children and Adolescents. Can. J. Cardiol..

[116] De Simone G., Richard B.D., Marcello C., Mary J.R., Elisa T.L., Helaine E.R., Barbara V.H. (2009). Metabolic Syndrome and Left Ventricular Hypertrophy in the Prediction of Cardiovascular Events-The Strong Heart Study. Nutr. Metab. Cardiovasc. Dis..

[117] Peterson L.R., Herrero P., Schechtman K.B., Racette S.B., Waggoner A.D., Kisrieva-Ware Z., Dence C., Klein S.B., Marsala J., Meyer T. (2004). Effect of Obesity and Insulin Resistance on Myocardial Substrate Metabolism and Efficiency in Young Women. Circulation.

[118] Borges-Canha M., Neves J.S., Libâni D., Von-Hafe M., Vale C., Araújo-Martins M., Leite A.R., Pimentel-Nunes P., Davide C., Adelino L.M. (2019). Association between Nonalcoholic Fatty Liver Disease and Cardiac Function and Structure—A Meta-Analysis. Endocrine.

[119] Kocabay G., Karabay C.Y., Colak Y., Oduncu V., Kalayci A., Akgun T., Guler A., Kirma C. (2014). Left Atrial Deformation Parameters in Patients with Non-Alcoholic Fatty Liver Disease: A 2D Speckle Tracking Imaging Study. Clin. Sci..

[120] Mondillo S., Cameli M., Caputo M.L., Lisi M., Palmerini E., Padeletti M., Ballo P. (2011). Early Detection of Left Atrial Strain Abnormalities by Speckle-Tracking in Hypertensive and Diabetic Patients with Normal Left Atrial Size. J. Am. Soc. Echocardiogr..

[121] Bekler A., Gazi E., Erbag G., Binnetoglu E., Barutcu A., Sen H., Temiz A., Altun B. (2015). Right Ventricular Function and Its Relationship with Grade of Hepatosteatosis in Non-Alcoholic Fatty Liver Disease. Cardiovasc. J. Afr..

[122] Anstee Q.M., Mantovani A., Tilg H., Targher G. (2018). Risk of Cardiomyopathy and Cardiac Arrhythmias in Patients with Nonalcoholic Fatty Liver Disease. Nat. Rev. Gastroenterol. Hepatol..

[123] Volzke H., Haring R., Lorbeer R., Wallaschofski H., Reffelmann T., Empen K., Rettig R., John U., Felix S.B., Dorr M. (2010). Heart Valve Sclerosis Predicts All-Cause and Cardiovascular Mortality. Atherosclerosis.

[124] Targher G., Day C.P., Bonora E. (2010). Risk of Cardiovascular Disease in Patients with Nonalcoholic Fatty Liver Disease. N. Engl. J. Med..

[125] Otto C.M., Kuusisto J., Reichenbach D.D., Gown A.M., O’Brien K.D. (1994). Characterization of the Early Lesion of ‘Degenerative’ Valvular Aortic Stenosis. Histological and Immunohistochemical Studies. Circulation.

[126] Goto T., Onuma T., Takebe K., Kral J.G. (1995). The Influence of Fatty Liver on Insulin Clearance and Insulin Resistance in Non-Diabetic Japanese Subjects. Int. J. Obes. Relat. Metab. Disord..

[127] Paulista M., Marcello R., Baumeister S.E., Dörr J.M., Wallaschofski H., Völzke H., Lieb W. (2013). Hepatic Steatosis Is Associated With Aortic Valve Sclerosis in the General Population The Study of Health in Pomerania (SHIP). Arterioscler. Thromb. Vasc. Biol..

[128] Paulus W.J., Tschope C. (2013). A Novel Paradigm for Heart Failure with Preserved Ejection Fraction: Comorbidities Drive Myocardial Dysfunction and Remodeling through Coronary Microvascular Endothelial Inflammation. J. Am. Coll. Cardiol..

[129] Belke D.D., Betuing S., Tuttle M.J., Graveleau C., Young M.E., Pham M., Zhang D., Cooksey R.C., McClain D.A., Litwin S.E. (2002). Insulin Signaling Coordinately Regulates Cardiac Size, Metabolism, and Contractile Protein Isoform Expression. J. Clin. Investig..

[130] Dhingra R., Gona P., Wang T.J., Fox C.S., D’Agostino R.B., Vasan R.S. (2010). Serum Gamma-Glutamyl Transferase and Risk of Heart Failure in the Community. Arter. Thromb. Vasc. Biol..

[131] Wannamethee S.G., Whincup P.H., Shaper A.G., Lennon L., Sattar N. (2012). Γ-Glutamyltransferase, Hepatic Enzymes, and Risk of Incident Heart Failure in Older Men. Arter. Thromb. Vasc. Biol..

[132] Emdin M., Pompella A., Paolicchi A. (2005). γ-Glutamyltransferase, Atherosclerosis, and Cardiovascular Disease: Triggering Oxidative Stress within the Plaque. Circulation.

[133] Hamaguchi M., Kojima T., Takeda N., Nagata C., Takeda J., Sarui H., Kawahito Y., Yoshida N., Suetsugu A., Kato T. (2007). Nonalcoholic Fatty Liver Disease Is a Novel Predictor of Cardiovascular Disease. World J. Gastroenterol..

[134] Abdeldyem S.M., Goda T., Khodeir S.A., Abou S.S., Abd-Elsalam S. (2017). Nonalcoholic Fatty Liver Disease in Patients with Acute Ischemic Stroke Is Associated with More Severe Stroke and Worse Outcome. J. Clin. Lipidol..

[135] El Hadi H., Di Vincenzo A., Vettor R., Rossato M. (2019). Cardio-Metabolic Disorders in Non-Alcoholic Fatty Liver Disease. Int. J. Mol. Sci..

[136] Bots M.L., Salonen J., Elwood P., Nikitin Y., Freire D., Inzitari D., Sivenius J., Trichopoulou A., Tuomilehto J., Koudstaal P. (2002). Gamma-Glutamyltransferase and Risk of Stroke: The EUROSTROKE Project. J. Epidemiol. Community Health.

[137] Paolicchi A., Emdin M., Ghliozeni E., Ciancia E., Passino C., Popoff G., Pompella A. (2004). Images in Cardiovascular Medicine, Human Atherosclerotic Plaques Contain Gamma-Glutamyl Transpeptidase Enzyme Activity. Circulation.

[138] Emdin M., Passino C., Franzini M., Paolicchi A., Pompella A. (2007). γ-Glutamyltransferase and Pathogenesis of Cardiovascular Diseases. Future Cardiol..

[139] Khalaf M.R., Hayhoe F.G. (1987). Cytochemistry of Gamma-Glutamyltransferase in Haemic Cells and Malignancies. Histochem. J..

[140] Stark A.A., Zeiger E., Pagano D.A. (1993). Glutathione Metabolism by Gamma-Glutamyltranspeptidase Leads to Lipid Peroxidation: Characterization of the System and Relevance to Hepatocarcinogenesis. Carcinogenesis.

[141] Stocker R., Keaney J., John F. (2004). Role of Oxidative Modifications in Atherosclerosis. Physiol. Rev..

[142] Ciavarella A., Gnocchi D., Custodero C., Lenato G.M., Fiore G., Sabbà C., Mazzocca A. (2021). Translational Insight into Prothrombotic State and Hypercoagulation in Nonalcoholic Fatty Liver Disease. Thromb. Res..

[143] Di Minno M.N.D., Tufano A., Rusolillo A., Di Minno G., Tarantino G. (2010). High Prevalence of Nonalcoholic Fatty Liver in Patients with Idiopathic Venous Thromboembolism. World J. Gastroenterol..

[144] Stine J., Jonathan G., Shah N.L., Argo C.K., Pelletier S.J., Caldwell S.H., Northup P.G. (2015). Northup Increased Risk of Portal Vein Thrombosis in Patients With Cirrhosis Due to Nonalcoholic Steatohepatitis. Liver Transpl..

[145] Nieuwdorp M., Stroes E.S., Meijers J.C., Buller H. (2005). Hypercoagulability in the Metabolic Syndrome. Curr. Opin. Pharmacol..

[146] Kim J.A., Kim J.E., Song S.H., Kim H.K. (2015). Influence of Blood Lipids on Global Coagulation Test Results. Ann. Lab. Med..

[147] Yun J.W., Cho Y.K., Park J.H., Kim H.J., Park D.I., Sohn C.I., Jeon W.K., Kim B.I. (2009). Abnormal Glucose Tolerance in Young Male Patients with Nonalcoholic Fatty Liver Disease. Liver Int..

[148] Undas A., Wiek I., Stepien E., Zmudka K., Tracz W. (2008). Hyperglycemia Is Associated with Enhanced Thrombin Formation, Platelet Activation, and Fibrin Clot Resistance to Lysis in Patients with Acute Coronary Syndrome. Diabetes Care.

[149] Vinik A.I., Erbas T., Park T.S., Nolan R., Pittenger G.L. (2001). Platelet Dysfunction in Type 2 Diabetes. Diabetes Care.

[150] Ceriello A. (1993). Coagulation Activation in Diabetes Mellitus: The Role of Hyperglycaemia and Therapeutic Prospects. Diabetologia.

[151] Mitropoulos K.A., Miller G.J., Watts G.F., Durrington P.N. (1992). Lipolysis of Triglyceride-Rich Lipoproteins Activates Coagulant Factor XII: A Study in Familial Lipoprotein-Lipase Deficiency. Atherosclerosis.

[152] Alderton W.K., Cooper C.E., Knowles R.G. (2001). Nitric Oxide Synthases: Structure, Function and Inhibition. Biochem. J..

[153] Dumas M.E., Barton R.H., Toye A., Cloarec O., Blancher C., Rothwell A., Fearnside J., Tatoud R., Blanc V., Lindon J.C. (2006). Metabolic Profiling Reveals a Contribution of Gut Microbiota to Fatty Liver Phenotype in Insulin-Resistant Mice. Proc. Natl. Acad. Sci. USA.

[154] Chen Y.M., Liu Y., Zhou R.F., Chen X.L., Wang C., Tan X.Y., Wang L.J., Zheng R.D., Zhang H.W., Ling W.H. (2016). Associations of Gut-Flora-Dependent Metabolite Trimethylamine-Noxide, Betaine and Choline with Non-Alcoholic Fatty Liver Disease in Adults. Sci. Rep..

[155] Zhu W., Gregory J.C., Org E., Buffa J.A., Gupta N., Wang Z., Li L., Fu X., Wu Y., Mehrabian M. (2016). Microbial Metabolite TMAO Enhances Platelet Hyperreactivity and Thrombosis Risk. Cell.

[156] Sookoian S., Castano G.O., Burgueno A.L., Rosselli M.S., Gianotti T.F., Mallardi P., Martino J.S., Pirola C.J. (2010). Circulating Levels and Hepatic Expression of Molecular Mediators of Atherosclerosis in Nonalcoholic Fatty Liver Disease. Atherosclerosis.

[157] Esmon C.T. (2005). The Interactions between Inflammation and Coagulation. Br. J. Haematol..

[158] Dole V.S., Bergmeier W., Patten I.S., Hirahashi J., Mayadas T.N., Wagner D.D. (2007). PSGL-1 Regulates Platelet P-Selectin-Mediated Endothelial Activation and Shedding of P-Selectin from Activated Platelets. Thromb. Haemost..

[159] Patil R., Sood G.K. (2017). Non-Alcoholic Fatty Liver Disease and Cardiovascular Risk. World J. Gastrointest. Pathophysiol..

[160] Abed H.S., Wittert G.A., Leong D.P., Shirazi M.G., Bahrami B., Middeldorp M.E., Lorimer M.F., Lau D.H., Antic N.A., Brooks A.G. (2013). Effect of Weight Reduction and Cardiometabolic Risk Factor Management on Symptom Burden and Severity in Patients with Atrial Fibrillation: A Randomized Clinical Trial. JAMA.

[161] Averna M. (2015). The Effect of Ezetimibe on NAFLD. Atheroscler. Suppl..

[162] Abe M., Matsuda M., Kobayashi H., Miyata Y., Nakayama Y., Komuro R., Fukuhara A., Shimomura I. (2008). Effects of Statins on Adipose Tissue Inflammation: Their Inhibitory Effect on MyD88-Independent IRF3/IFN-Beta Pathway in Macrophages. Arter. Thromb. Vasc. Biol..

[163] Parisi V., Petraglia L., D’Esposito V., Cabaro S., Rengo G., Caruso A., Grimaldi M.G., Baldascino F., De Bellis A., Vitale D. (2019). Therapy Modulates Thickness and Inflammatory Profile of Human Epicardial Adipose Tissue. Int. J. Cardiol..

[164] Li Y.D., Tang B.P., Guo F., Li J.X., Han W., Tang Q., Zhang Y.Y. (2013). Effect of Atorvastatin on Left Atrial Function of Patients with Paroxysmal Atrial Fibrillation. Genet. Mol. Res..

[165] Fauchier L., Pierre B., de Labriolle A., Grimard C., Zannad N., Babuty D. (2008). Antiarrhythmic Effect of Statin Therapy and Atrial Fibrillation a Meta-Analysis of Randomized Controlled Trials. J. Am. Coll. Cardiol..

[166] Kim J., Lee H., An J., Song Y., Lee C.K., Kim K., Kong H. (2019). Alterations in Gut Microbiota by Statin Therapy and Possible Intermediate Effects on Hyperglycemia and Hyperlipidemia. Front. Microbiol..

[167] DeFronzo R.A., Tripathy D., Schwenke D.C., Banerji M.A., Bray G.A., Buchanan T.A., Clement S.C., Henry R.R., Hodis H.N., Kitabchi A.E. (2011). Pioglitazone for Diabetes Prevention in Ipaired Glucose Tolerance. N. Engl. J. Med..

[168] Dormandy J.A., Charbonnel B., Eckland D.J.A., Erdmann E., Massi-Benedetti M., Moules I.K., Skene A.M., Tan M.H., Lefebvre P.J., Murray G.D. (2005). Secondary Prevention of MacroVascular Events in Patients with Type 2 Diabetes in the Proactive Study (Prospective PioglitAzone Clinical Trial in MacroVascular Events): A Randomised Controlled Trial. Lancet.

[169] Musso G., Cassader M., Paschetta E., Gambino R. (2017). Thiazolidinediones and Advanced Liver Fibrosis in Nonalcoholic Steatohepatitis: A Meta-Analysis. JAMA Intern. Med..

[170] Li J.M., Yu R., Zhang L.P., Wen S.Y., Wang S.J., Zhang X.Y., Xu Q., Kong L.D. (2019). Dietary Fructose-Induced Gut Dysbiosis Promotes Mouse Hippocampal Neuroinflammation: A Benefit of Short-Chain Fatty Acids. Microbiome.

[171] Stumvoll M., Nurjhan N., Perriello G., Dailey G., Gerich J.E. (1995). Metabolic Effects of Metformin in Non-Insulin-Dependent Diabetes Mellitus. N. Engl. J. Med..

[172] Lomba R., Lutchman G., Kleiner D.E., Ricks M., Feld J.J., Borg B.B., Modi A., Nagabhyru P., Sumner A.E., Liang T.J. (2009). Clinical Trial: Pilot Study of Metformin for the Treatment of Non-Alcoholic Steatohepatitis. Aliment. Pharmacol. Ther..

[173] He L., Sabet A., Djedjos S., Miller R., Sun X., Hussain M.A., Radovick S., Wondisford F.E. (2009). Metformin and Insulin Suppress Hepatic Gluconeogenesis through Phosphorylation of CREB Binding Protein. Cell.

[174] Kohjima M., Higuchi N., Kato M., Koroh K., Yoshimoto T., Fujino T., Yada R., Yada R., Harada N., Enjoji M. (2008). SREBP-1c, Regulated by the Insulin and AMPK Signaling Pathways, Plays a Role in Nonalcoholic Fatty Liver Disease. Int. J. Mol. Med..

[175] Nair S., Diehl A.M., Wiseman M., Farr G.H., Perrillo R.P. (2004). Metformin in the Treatment of Non-Alcoholic Steatohepatitis: A Pilot Open Label Trial. Aliment. Pharmacol. Ther..

[176] Bugianesi E., Gentilcore E., Manini R., Natale S., Vanni E., Vilanova N., David E., Rizzetto M., Marchesini G. (2005). A Randomized Controlled Trial of Metformin versus Vitamin E or Prescriptive Diet in Nonalcoholic Fatty Liver Disease. Am. J. Gastroenterol..

[177] De Oliveira C.P.M.S., Stefano J.T., De Siqueira E.R.F., Soares Silva L., Ferraz de Campos Marzo D., Lima V.M.R., Furuya C.K., Mello E.S., Souza F.G., Rabello F. (2008). Combination of N-Acetylcysteine and Metformin Improves Histological Steatosis and Fibrosis in Patients with Non-Alcoholic Steatohepatitis. Hepatol. Res..

[178] Idilman R., Mizrak D., Corapcioglu D., Bektas M., Doganay B., Sayki M., Coban S., Erden E., Soykan I., Emral R. (2008). Clinical Trial: Insulin-Sensitizing Agents May Reduce Consequences of Insulin Resistance in Individuals with Non-Alcoholic Steatohepatitis. Aliment. Pharmacol. Ther..

[179] Janiec D.J., Jacobson E.R., Freeth A., Spaulding L., Blaszyk H. (2005). Histologic Variation of Grade and Stage of Non-Alcoholic Fatty Liver Disease in Liver Biopsies. Obes. Surg..

[180] Haukeland J.W., Konopski Z., Eggesb H.B., von Volkmann H.L., Raschpichler G., Bjoro K., Haaland T., Loberg E.M., Birkeland K. (2009). Metformin in Patients with Non-Alcoholic Fatty Liver Disease: A Randomized, Controlled Trial. Scand. J. Gastroenterol..

[181] Omer Z., Cetinkalp S., Akyildiz M., Yilmaz F., Batur Y., Yilmaz C., Akarca U. (2010). Efficacy of Insulin-Sensitizing Agents in Nonalcoholic Fatty Liver Disease. Eur. J. Gastroenterol. Hepatol..

[182] Nar A., Gedik O. (2009). The Effect of Metformin on Leptin in Obese Patients with Type 2 Diabetes Mellitus and Nonalcoholic Fatty Liver Disease. Acta Diabetol..

[183] Loria P., Lonardo A., Bellentani S., Day C.P., Marchesini G., Carulli N. (2007). Non-Alcoholic Fatty Liver Disease (NAFLD) and Cardiovascular Disease: An Open Question. Nutr. Metab. Cardiovasc. Dis..

[184] Kirpichnikov D., McFarlane S.I., Sowers J.R. (2002). Metformin: An Update. Ann. Intern. Med..

[185] Morrow V.A., Foufelle F., Connell J.M.C., Petrie J.R., Gould G.W., Salt I.P. (2003). Direct Activation of AMP-Activated Protein Kinase Stimulates Nitric-Oxide Synthesis in Human Aortic Endothelial Cells. J. Biol. Chem..

[186] Mantovani A., Byrne C.D., Scorletti E., Mantzoros C.S., Targher G. (2020). Efficacy and Safety of Anti-Hyperglycaemic Drugs in Patients with Non-Alcoholic Fatty Liver Disease with or without Diabetes: An Updated Systematic Review of Randomized Controlled Trials. Diabetes Metab..

[187] Zhu J., Yu X., Zheng Y., Li J., Wang Y., Lin Y., He Z., Zhao W., Chen C., Qiu K. (2020). Association of Glucose-Lowering Medications with Cardiovascular Outcomes: An Umbrella Review and Evidence MAP. Lancet Diabetes Endocrinol..

[188] Nakamura H., Niwano S., Niwano H., Fukaya H., Murakami M., Kishihara J., Satoh A., Yoshizawa T., Ishizue N., Igarashi T. (2019). Liraglutide Suppresses Atrial Electrophysiological Changes. Heart Vessel..

[189] Morigny P., Houssier M., Mouisel E., Langin D. (2016). Adipocyte Lipolysis and Insulin Resistance. Biochimie.

[190] Armstrong M.J., Hazlehurst J.M., Hull D., Guo K., Borrows S., Yu J., Gough S.C., Newsome P.N., Tomlinson J.W. (2014). Abdominal Subcutaneous Adipose Tissue Insulin Resistance and Lipolysis in Patients with Non-alcoholic Steatohepatitis. Diabetes Obes. Metab..

[191] Sindhu S., Thomas R., Shihab P., Sriraman D., Behbehani K., Ahmad R. (2015). Obesity Is a Positive Modulator of IL-6R and IL-6 Expression in the Subcutaneous Adipose Tissue: Significance for Metabolic Inflammation. PLoS ONE.

[192] Cnop M., Havel P.J., Utzschneider K.M., Carr D.B., Sinha M.K., Boyko E.J., Retzlaff B.M., Knopp R.H., Brunzell J.D., Kahn S.E. (2003). Relationship of Adiponectin to Body Fat Distribution, Insulin Sensitivity and Plasma Lipoproteins: Evidence for Independent Roles of Age and Sex. Diabetologia.

[193] Yamauchi T., Kamon J., Minokoshi Y., Ito Y., Waki H., Uchida S., Yamashita S., Noda M., Kita S., Ueki K. (2002). Adiponectin Stimulates Glucose Utilization and Fatty-acid Oxidation by Activating AMP-activated Protein Kinase. Nat. Med..

[194] Khan R.S., Bril F., Cusi K., Newsome P.N. (2019). Modulation of Insulin Resistance in Nonalcoholic Fatty Liver Disease. Hepatology.

[195] Hui J.M., Hodge A., Farrell G.C., Kench J.G., Kriketos A., George J. (2004). Beyond Insulin Resistance in NASH: TNF-alpha or Adiponectin?. Hepatology.

[196] Perry R.J., Samuel V.T., Petersen K.F., Shulman G.I. (2014). The Role of Hepatic Lipids in Hepatic Insulin Resistance and Type 2 Diabetes. Nature.

[197] Tian L., Jin T. (2016). The Incretin Hormone GLP-1 and Mechanisms Underlying Its Secretion. J. Diabetes.

[198] Yaribeygi H., Sathyapalan T., Sahebkar A. (2019). Molecular Mechanisms by Which GLP-1 RA and DPP-4i Induce Insulin Sensitivity. Life Sci..

[199] Svegliati-Baroni G., Saccomanno S., Rychlicki C., Agostinelli L., De Minicis S., Candelaresi C., Faraci G., Pacetti D., Vivarelli M., Nicolini D. (2011). Glucagon-like Peptide-1 Receptor Activation Stimulates Hepatic Lipid Oxidation and Restores Hepatic Signalling Alteration Induced by a High-fat Diet in Nonalcoholic Steatohepatitis. Liver Int..

[200] Hazlehurst J.M., Woods C., Marjot T., Cobbold J.F., Tomlinson J.W. (2016). Non-alcoholic Fatty Liver Disease and Diabetes. Metabolism.

[201] Klonoff DC B.J., Nielsen L.L., Guan X., Bowlus C.L., Holcombe J.H., Wintle M.E., Maggs D.G. (2007). Metabolic Effects of Two Years of Exenatide Treatment on Diabetes, Obesity, and Hepatic Biomarkers in Patients with Type 2 Diabetes: An Interim Analysis of Data from the Open-label, Uncontrolled Extension of Three Double-blind, Placebo-controlled Trials. Clin. Ther..

[202] Klonoff D.C., Buse J.B., Nielsen L.L., Guan X., Bowlus C.L., Holcombe J.H., Wintle M.E., Maggs D.G. (2008). Exenatide Effects on Diabetes, Obesity, Cardiovascular Risk Factors and Hepatic Biomarkers in Patients with Type 2 Diabetes Treated for at Least 3 Years. Curr. Med. Res. Opin..

[203] Castera L., Vilgrain V., Angulo P. (2013). Noninvasive Evaluation of NAFLD. Nat. Rev. Gastroenterol. Hepatol..

[204] Vuppalanchi R., Siddiqui M.S., Van Natta M.L., Hallinan E., Brandman D., Kowdley K., Neuschwander-Tetri B.A., Loomba R., Dasarathy S., Abdelmalek M. (2018). Performance Characteristics of Vibration-controlled Transient Elastography for Evaluation of Nonalcoholic Fatty Liver Disease. Hepatology.

[205] Dong Y., Lv Q., Li S., Wu Y., Li L., Zhang F., Sun X., Tong N. (2017). Efficacy and Safety of Glucagon-like Peptide-1 Receptor Agonists in Non-alcoholic Fatty Liver Disease: A Systematic Review and Meta-analysis. Clin. Res. Hepatol. Gastroenterol..

[206] Lv X., Dong Y., Hu L., Lu F., Zhou C., Qin S. (2020). Glucagon-like Peptide-1 Receptor Agonists (GLP-1 RAs) for the Management of Nonalcoholic Fatty Liver Disease (NAFLD): A Systematic Review. Endocrinol. Diab. Metab..

[207] Sattar N., Fitchett D., Hantel S., George J.T., Zinman B. (2018). Empagliflozin Is Associated with Improvements in Liver Enzymes Potentially Consistent with Reductions in Liver Fat: Results from Randomised Trials Including the EMPA-REG OUTCOME^®^ Trial. Diabetologia.

[208] Nakano S., Katsuno K., Isaji M., Nagasawa T., Buehrer B., Walker S., Wilkison W.O., Cheatham B. (2015). Remogliflozin Etabonate Improves Fatty Liver Disease in Diet-Induced Obese Male Mice. J. Clin. Exp. Hepatol..

[209] Komiya C., Tsuchiya K., Shiba K., Miyachi Y., Furuke S., Shimazu N., Yamaguchi S., Kanno K., Ogawa Y. (2016). Ipragliflozin Improves Hepatic Steatosis in Obese Mice and Liver Dysfunction in Type 2 Diabetic Patients Irrespective of Body Weight Reduction. PLoS ONE.

[210] Shibuya T., Fushimi N., Kawai M., Yoshida Y., Hachiya H., Ito S., Kawai H., Ohashi N., Mori A. (2018). Luseogliflozin Improves Liver Fat Deposition Compared to Metformin in Type 2 Diabetes Patients with Non-Alcoholic Fatty Liver Disease: A Prospective Randomized Controlled Pilot Study. Diabetes Obes. Metab..

[211] Jojima T., Tomotsune T., Iijima T., Akimoto K., Suzuki K., Aso Y. (2016). Empagliflozin (an SGLT2 Inhibitor), Alone or in Combination with Linagliptin (a DPP-4 Inhibitor), Prevents Steatohepatitis in a Novel Mouse Model of Non-Alcoholic Steatohepatitis and Diabetes. Diabetol. Metab. Syndr..

[212] Seko Y., Sumida Y., Tanaka S., Mori K., Taketani H., Ishiba H., Hara T., Okajima A., Umemura A., Nishikawa T. (2017). Effect of Sodium Glucose Cotransporter 2 Inhibitor on Liver Function Tests in Japanese Patients with Non-Alcoholic Fatty Liver Disease and Type 2 Diabetes Mellitus. Hepatol. Res..

[213] Sumida Y., Murotani K., Saito M., Tamasawa A., Osonoi Y., Yoneda M., Osonoi T. (2019). Effect of Luseogliflozin on Hepatic Fat Content in Type 2 Diabetes Patients with Non-Alcoholic Fatty Liver Disease: A Prospective, Single-Arm Trial (LEAD Trial). Hepatol. Res..

[214] Eriksson J.W., Lundkvist P., Jansson P.A., Johansson L., Kvarnström M., Moris L., Miliotis T., Forsberg G.B., Risérus U., Lind L. (2018). Effects of Dapagliflozin and N-3 Carboxylic Acids on Non-Alcoholic Fatty Liver Disease in People with Type 2 Diabetes: A Double-Blind Randomised Placebo-Controlled Study. Diabetologia.

[215] Calzadilla Bertot L., Adams L.A. (2016). The Natural Course of Non-Alcoholic Fatty Liver Disease. Int. J. Mol. Sci..

[216] Shah A.G., Lydecker A., Murray K., Tetri B.N., Contos M.J., Sanyal A.J. (2009). NASH Clinical Research Network. Comparison of Noninvasive Markers of Fibrosis in Patients with Nonalcoholic Fatty Liver Disease. Clin. Gastroenterol. Hepatol..

[217] Angulo P., Hui J.M., Marchesini G., Bugianesi E., George J., Farrell G.C., Enders F., Saksena S., Burt A.D., Bida J.P. (2007). The NAFLD Fibrosis Score: A Noninvasive System That Identifies Liver Fibrosis in Patients with NAFLD. Hepatology.

[218] Tobita H., Sato S., Miyake T., Ishihara S., Kinoshita Y. (2017). Effects of Dapagliflozin on Body Composition and Liver Tests in Patients with Nonalcoholic Steatohepatitis Associated with Type 2 Diabetes Mellitus: A Prospective, Open-Label, Uncontrolled Study. Curr. Ther. Res. Clin. Exp..

[219] Musso G., Cassader M., Rosina F., Gambino R. (2012). Impact of Current Treatments on Liver Disease, Glucose Metabolism and Cardiovascular Risk in Non-Alcoholic Fatty Liver Disease (NAFLD): A Systematic Review and Meta-Analysis of Randomized Trials. Diabetologia.

[220] Yamashita H., Takenoshita M., Sakurai M., Bruick R.K., Henzel W.J., Shillinglaw W., Arnot D., Uyeda K. (2001). A Glucose-Responsive Transcription Factor That Regulates Carbohydrate Metabolism in the Liver. Proc. Natl. Acad. Sci. USA.

[221] Shimizu M., Suzuki K., Kato K., Jojima T., Iijima T., Murohisa T., Iijima M., Takekawa H., Usui I., Hiraishi H. (2019). Evaluation of the Effects of Dapagliflozin, a Sodium-Glucose Co-Transporter-2 Inhibitor, on Hepatic Steatosis and Fibrosis Using Transient Elastography in Patients with Type 2 Diabetes and Non-Alcoholic Fatty Liver Disease. Diabetes Obes. Metab..

[222] Cotter D.G., Ercal B., Huang X., Leid J.M., d’Avignon D.A., Graham M.J., Dietzen D.J., Brunt E.M., Patti G.J., Crawford P.A. (2014). Ketogenesis Prevents Diet-Induced Fatty Liver Injury and Hyperglycemia. J. Clin. Investig..

[223] Akuta N., Kawamura Y., Fujiyama S., Sezaki H., Hosaka T., Kobayashi M., Kobayashi M., Saitoh S., Suzuki F., Suzuki Y. (2020). SGLT2 Inhibitor Treatment Outcome in Nonalcoholic Fatty Liver Disease Complicated with Diabetes Mellitus: The Long-Term Effects on Clinical Features and Liver Histopathology. Intern. Med..

[224] Oikonomoua D., Georgiopoulosb G., Katsib V., Kourekb C., Tsioufisb C., Alexopouloub A., Koutlic E., Tousoulisb D. (2018). Non-Alcoholic Fatty Liver Disease and Hypertension: Coprevalent or Correlated?. Eur. J. Gastroenterol. Hepatol..

[225] Kaji K., Yoshiji H., Kitade M., Ikenaka Y., Noguchi R., Shirai Y., Aihara Y., Namisaki T., Yoshii J., Yanase K. (2011). Combination Treatment of Angiotensin II Type I Receptor Blocker and New Oral Iron Chelator Attenuates Progression of Nonalcoholic Steatohepatitis in Rats. Am. J. Physiol. Gastrointest. Liver Physiol..

[226] Georgescu E.F., Ionescu R., Niculescu M., Mogoanta L., Vancica L. (2009). Angiotensin-Receptor Blockers as Therapy for Mild-to-Moderate Hypertension-Associated Non-Alcoholic Steatohepatitis. World J. Gastroenterol..

[227] Hirata T., Tomita K., Kawai T., Yokoyama H., Shimada A., Kikuchi M., Hirose H., Ebinuma H., Irie J., Ojiro K. (2013). Effect of Telmisartan or Losartan for Treatment of Nonalcoholic Fatty Liver Disease: Fatty Liver Protection Trial by Telmisartan or Losartan Study (FANTASY). Int. J. Endocrinol..

[228] Randomized Global Phase 3 Study to Evaluate the Impact on NASH with Fibrosis of Obeticholic Acid Treatment (REGENERATE) U.S. National Library of Medicine. Clinical Trials.gov. https://clinicaltrials.gov/ct2/show/NCT02548351.

[229] Sanyal A., Charles E.D., Neuschwander-Tetri B.A., Loomba R., Harrison S.A., Abdelmalek M.F., Lawitz E.J., Halegoua-DeMarzio D., Kundu S., Noviello S. (2019). Pegbelfermin (BMS-986036), a PEGylated Fibroblast Growth Factor 21 Analogue, in Patients with Non-Alcoholic Steatohepatitis: A Randomised, Double-Blind, Placebo-Controlled, Phase 2a Trial. Lancet.

[230] Friedman S.L., Ratziu V., Harrison S.A., Abdelmalek M.F., Aithal G.P., Caballeria J., Francque S., Farrell G., Kowdley K.V., Craxi A. (2018). A Randomized, Placebo-Controlled Trial of Cenicriviroc for Treatment of Nonalcoholic Steatohepatitis with Fibrosis. Hepatology.

